# Bottom-up engineering of the nucleus pulposus using a photocrosslinkable decellularized matrix hydrogel attenuates inflammaging and enhances microtissue-mediated regeneration

**DOI:** 10.1016/j.mtbio.2025.102347

**Published:** 2025-09-24

**Authors:** Xiaoxiao Li, Xiangwei Li, Dandan Zhou, Yanqin Xu, Biemin Sun, Yanzhu Hu, Yibo Zhu, Junxian Hu, Zeyu Pang, Chen Zhao, Yongjian Gao, You Long, Pei Li, Qiang Zhou, Yiyang Wang

**Affiliations:** a Department of Orthopedics, The Third Affiliated Hospital of Chongqing Medical University, Chongqing, 401120, China; b Tissue Repairing and Biotechnology Research Center, The Third Affiliated Hospital of Chongqing Medical University, Chongqing, 401120, China; c Department of Geriatric Medicine, Jiulongpo People's Hospital of Chongqing, Chongqing, 400050, China; d College of Chemistry and Chemical Engineering, Chongqing University, Chongqing, 400044, China; e Department of Surgery, TUM School of Medicine and Health, Klinikum rechts der Isar, Technical University of Munich, Munich, 81675, Germany

**Keywords:** Nucleus pulposus progenitor cells (NPPCs), Photocrosslinkable hydrogel, Microtissue, Tissue engineered nucleus pulposus (TE-NP), Inflammaging

## Abstract

Degenerative disc disease (DDD), characterized by the pathological deterioration of nucleus pulposus (NP) tissue, affects millions globally. Tissue engineering strategies offer potential to create tissue-engineered NP (TE-NP) analogs to address DDD. However, traditional "top-down' approaches face challenges in achieving uniform cell distribution and replicating the intradiscal extracellular matrix (ECM) environment. In contrast, a "bottom-up' strategy utilizing microscale seed units represents a promising alternative. This study introduces an innovative "bottom-up' approach for constructing TE-NP, leveraging bioreactor-cultivated NP microtissues (NP-MTs) as seed units and a novel methacrylate-modified decellularized nucleus pulposus matrix (DNPM-MA) hydrogel as a supporting biomaterial. NP-MTs cultivated under low-magnitude hydrostatic pressure exhibit nascent ECM surroundings adapting well to the intradiscal microenvironment. The DNPM-MA hydrogel, with its compositional and mechanical attributes, supports the growth, migration, proliferation, and ECM synthesis of NP-MTs, making it an ideal biomaterial for long-term cultivation. The combination of NP-MTs and the DNPM-MA hydrogel yielded superior tissue regeneration outcomes both in vitro and in vivo. Transcriptome and molecular assessments revealed a correlation between the biological properties of the DNPM-MA hydrogel and the attenuation of inflammaging within encapsulated NP-MTs. Overall, this innovative "bottom-up' constructed TE-NP exhibits superior regenerative potential and is a promising tissue engineering strategy for treating DDD.

## Graphical abstract

**Figure:**
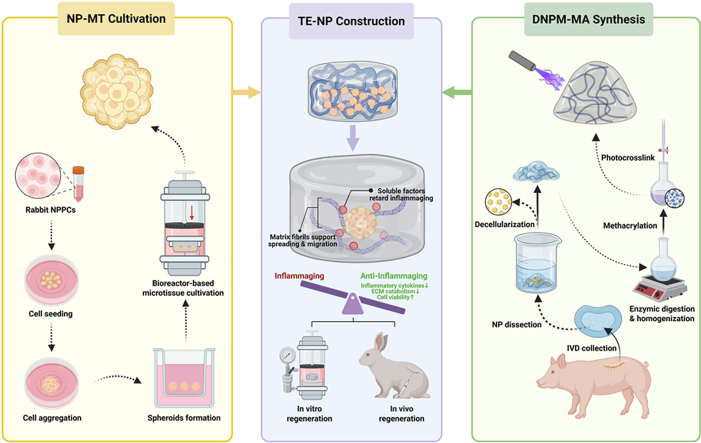

## 1 Introduction

Degenerative disc disease (DDD) has become a principal causative factor of global disability, predominantly due to its role as the most common cause of low back pain (LBP) and other severe spinal disorders [1]. Previous investigations have suggested that the local inflammatory response within the nucleus pulposus (NP), the central component of the intervertebral disc (IVD), represents a key pathological alteration driving the progression of disc degeneration [2,3]. Additionally, the NP is classified as an immune-privileged organ; thus, tissue-engineered nucleus pulposus (TE-NP) analogs have the potential to be utilized as readily available substitutes for allogeneic transplantation [4].

Since its emergence in the 1980s, the discipline of tissue engineering has experienced ongoing refinements in methodologies and conceptual frameworks [5]. Specifically, the contemporary ascent of "bottom-up' tissue engineering, involving the cultivation of microscale seed units, such as cell aggregates, microtissues, and organoids, has introduced an innovative paradigm for the fabrication of engineered analogs and organs [[6], [7], [8]]. Our previous study applied a three-dimensional (3D) spheroid culture technique to fabricate nucleus pulposus cell spheroids as the seed units for TE-NP construction [9]. These cell spheroids exhibited superior efficacy in tissue regeneration compared to TE-NP derived from isolated seed cells [9]. Clinical research has also indicated that the intradiscal transplantation of stem cell spheroids is a safe and viable therapeutic option for treating discogenic LBP [10]. However, in a subsequent investigation, we found that the regenerated tissues derived from nucleus pulposus cell spheroid-based TE-NP showed certain disparities in extracellular matrix (ECM) composition compared to those derived from healthy NP tissues [9]. Specifically, the regenerated tissues displayed a fibril-like structure with high expression of collagen type I (Col I), in contrast to healthy, hydrated NP tissues characterized by abundant collagen type II (Col II), glycosaminoglycans (GAGs) and proteoglycans (PGs) [9]. In essence, the functional ECM homeostasis of NP tissues was not effectively restored. Hence, restoring functional ECM homeostasis is pivotal not only for refining the construction of TE-NP, but also for improving the regenerative and repair outcomes of degenerated NP tissue [11,12].

The mature IVD is composed of three main structures: the core NP, surrounding annulus fibrosus, and the end plate that adjoins the IVD and vertebrae [13]. The NP is an important component of the IVD that is responsible for stress-absorbing functions and for preserving the flexibility of the spinal motion segment [14,15]. The biological characterizations of the NP tissue are mainly determined by the gelatinous ECM, which is composed of a collagen network (mainly Col II) in conjunction with hydrophilic constituents, such as PGs (mainly aggrecan, ACAN) and GAGs [16,17]. The functional ECM of NP tissue provides a structural framework for cellular adhesion and plays an indispensable role in foundational biological processes, including cell-to-cell and cell-to-ECM signaling [11,17]. An aberrant increase in the fibrotic intensity of the NP matrix can trigger a vicious cycle between diminished cellular bioactivity and the exacerbation of ECM homeostasis, which ultimately leads to the impairment of its inherent biological and mechanical functions [17,18]. Therefore, further improvement of functional ECM homeostasis in regenerated NP represents a crucial challenge that urgently needs to be addressed in the current stage of TE-NP construction. Promising optimization strategies involve the cultivation of microscale seed units derived from progenitor/stem cells and the advanced refinement of bioscaffold design and functionality [5,6,19].

**Fig. 1:**
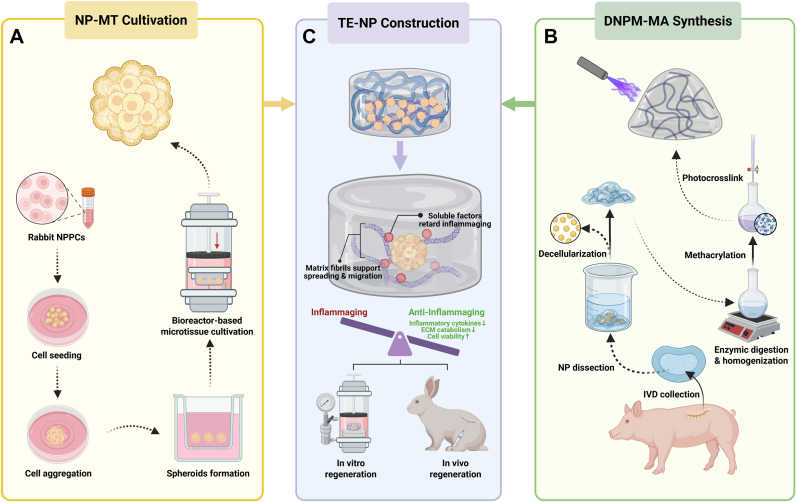
Schematic diagram of the study design. (A) Cultivation of NP-MT using a hydrostatic bioreactor. (B) Synthesis of the novel photocrosslinkable DNPM-MA hydrogel. (C) Construction of TE-NP using NP-MTs as seed units and the DNPM-MA hydrogel as a supporting biomaterial.

Here, we introduce an innovative “bottom-up” strategy for TE-NP construction based on microtissues and a novel photocrosslinkable decellularized nucleus pulposus matrix (DNPM)-based hydrogel. The nucleus pulposus microtissues (NP-MTs) were cultivated using nucleus pulposus progenitor cells (NPPCs) in a self-designed hydrostatic bioreactor (patent No. ZL 202120535745.3). Furthermore, a novel photocrosslinkable hydrogel, methacrylate-modified decellularized nucleus pulposus matrix (DNPM-MA) hydrogel, was developed to serve as a scaffold for the integration of NP-MTs and subsequently to construct the TE-NP. Our study investigated the biological impact of gradient hydrostatic pressures within the low-magnitude range on the cultivation of NP-MTs and elucidated conducive pressure stress parameters to promote the optimal development of NP-MTs. The optimal synthetic parameters and concentration of DNPM-MA hydrogel for the biomimetic construction of TE-NP were determined through assessments involving quantitative proteomics, ^1^H NMR spectroscopy, biocompatibility, and mechanical tests. The in vitro and in vivo tissue regeneration efficacy of the TE-NP constructed using NP-MTs as the seed units and the DNPM-MA hydrogel as the scaffold were further evaluated via radiological and histological assessments. Additionally, RNA sequencing and molecular biology experiments were performed to elucidate the regulatory mechanisms involved in the regeneration process induced by the DNPM-MA hydrogel. On the one hand, our findings suggested that the cells in the NP-MTs were encapsulated within a nascent ECM, enhancing their adaptability to the intradiscal microenvironment. Concurrently, the DNPM-MA hydrogel, enriched with NP matrix components, could retard inflammation-associated senescence of the laden microtissues and exhibited dual biomimetic characteristics, emulating both the structural properties and functional components of the native NP matrix (Fig. 1).

## 2 Materials and methods

### 2.1 Materials

Tris-HCl buffer was purchased from Shanghai Sinopharm Chemical Reagent Co., Ltd. Triton X-100, ethylenediaminetetraacetic acid (EDTA), aprotinin, and lithium phenyl-2,4,6-trimethylbenzoylphosphinate (LAP) were purchased from Shanghai Aladdin Biochemical Technology Co., Ltd. Ribonuclease (RNase) and deoxyribonuclease (DNase) were purchased from Beijing Solarbio Biotechnology Co., Ltd. DNA quantification assay kits were purchased from Invitrogen, Thermo Fisher Scientific Inc., Waltham, Massachusetts, USA. Hematoxylin eosin (HE), Alizarin Red, Oil Red O, Alcian blue, Safranin O, and Sirius red staining reagents were purchased from Wuhan Servicebio Biotechnology Co., Ltd. Glycosaminoglycan (GAG) and collagen type II (Col II) ELISA kits were purchased from Quanzhou Jiubang Biotechnology Co., Ltd. Live/dead staining kits, F-actin and diamidino-phenylindole (DAPI) dual fluorescence staining, and alkene-coupled hydrogel fluorescent dyes were purchased from Hangzhou EFL Biochemical Technology Co., Ltd. DMEM/F12 and penicillin/streptomycin were purchased from Cytiva Inc., Medford, Massachusetts, USA. Fetal bovine serum (FBS) was from Gibco, Thermo Fisher Scientific Inc., USA. Antibodies against NADPH oxidase 4 (Nox4), CD73 and CD105 were purchased from Novus Biologicals, LLC, Littleton, Colorado, USA. Antibodies against interleukin-1 beta (IL1β), aggrecan (ACAN), Col II, matrix metalloproteinase 9 (MMP9), matrix metalloproteinase 13 (MMP13), and CD34 were purchased from Wuhan Proteintech Biotechnology Co., Ltd. Antibody against HLA-DR was purchased from from Zenbio, Inc., Houston, Texas, USA. Antibodies against ASK1 and Phospho-p38 (p-p38) were purchased from Affinity Biosciences Co., Ltd., USA. Osteogenic, adipogenic, and chondrogenic differentiation media were purchased from Guangzhou OriCell Biosciences Co., Ltd. Protein extraction, senescence-associated β-galactosidase (SA-β-gal) assay kits, and reactive oxygen species (ROS) 2′,7′-dichlorofluorescein diacetate (DCFH-DA) assay kit were purchased from Xiamen Beyotime Biotechnology Co., Ltd. In addition, were purchased from Beyotime, China. New Zealand rabbits, and nude mice were obtained from the Animal Center of Chongqing Medical University (CQMU) and all animal procedures were performed after receiving approval from the Institutional Animal Care and Use Committee (IACUC) of CQMU (IACUC-CQMU-2023-0148).

### 2.2 Preparation of DNPM

Porcine lumbar IVDs were obtained from a local slaughterhouse. The overlying endplate and surrounding annulus fibrosus tissues were meticulously excised. The NP tissues were then dissected into fragments and immersed in a detergent solution containing 10 mM Tris-HCl buffer, 3 % Triton X-100, 0.1 % EDTA, and 10 KIU/ml aprotinin for 48 h at room temperature. Subsequently, the samples were treated with a nuclease solution of 10 mM Tris-HCl buffer, 0.2 mg/ml RNase, and 0.2 mg/ml DNase for 48 h at 37 °C to remove nucleic acids.

### 2.3 Component analysis of DNPM

To evaluate cell elimination, fresh and decellularized NP specimens were embedded in paraffin, sectioned into 5 μm slices, and stained with HE and DAPI. The tissue specimens were weighed to record the wet weight and digested overnight at 60 °C in a phosphate-buffered EDTA solution. The DNA content was then quantified utilizing a total DNA quantification assay following the manufacturer's instructions.

To assess major ECM components, GAG and total collagen expression in fresh and decellularized NP specimens were analyzed using Alcian blue and Sirius red staining, as described in previous studies [12,20]. In addition, a quantitative analysis of GAG and Col II levels was performed using ELISA kits according to the manufacturer's instructions.

### 2.4 Preparation of the DNPM-MA hydrogel

Decellularized NP samples were lyophilized and ground into DNPM powders. Gelation was achieved using a modified "Freytes method': DNPM powders were resuspended in a digestion solution composed of 0.1 M hydrochloric acid and 0.2 % porcine pepsin and homogenized for 1 h at room temperature. A solution consisting of 10 mM Tris-HCl buffer with 0.1 mM sodium hydroxide was then used to neutralize the solution, resulting in a gelatinous DNPM fluid. After dialysis against deionized water for 3 days, the DNPM fluid was lyophilized into cotton-like powders.

To synthesize DNPM-MA hydrogels, 0.5 g of DNPM powder was dissolved in deionized water, and 0.5 ml of methacrylic anhydride was added dropwise to the solution in an ice bath. The pH was maintained between 8 and 10 with 0.1 mM sodium hydroxide, and the reaction proceeded overnight in the dark at 4 °C. After centrifugation and pH adjustment to 7.4 with hydrochloric acid, the product was dialyzed for 3 days and then frozen and lyophilized into a hydrogel precursor.

### 2.5 Characterization of the DNPM-MA hydrogel

The degree of methacrylation was analyzed using ^1^H NMR spectroscopy (JEOL, Japan), as described in a previous study [21]. Specifically, the integrated area of the lysine methylene protons (δ = 2.9–3.1 ppm), which corresponds to the primary amine group, was compared between DNPM and DNPM-MA. The MA substitution degree (%) was calculated using the following formula: (1-A_DNPM-MA_/A_DNPM_) × 100 %.

To further confirm the successful incorporation of methacrylate groups, Fourier Transform Infrared (FTIR) spectroscopy was conducted. The appearance of characteristic peaks corresponding to C=C stretching vibrations verified the presence of methacrylate moieties in the modified hydrogel.

In addition, the quantitative proteomic analysis identified differentially expressed proteins (DEPs) among fresh NP, DNPM, and DNPM-MA, and a Gene Ontology (GO) enrichment analysis of DEPs was applied to explore potential pathways of regulating the materials’ biological functions.

### 2.6 Photocrosslink of the DNPM-MA hydrogel

Hydrogel precursors of DNPM-MA and 0.25 % w/v LAP were mixed and dissolved in PBS at 37 °C. The fully dissolved hydrogel precursor solution was then divided for various study applications and crosslinked under UV irradiation (365 nm, 850 mW) for 30 s at a distance of about 10 cm. For alternative crosslinking conditions, precursor solutions containing DNPM-MA and 0.15 % (w/v) LAP were exposed to visible light (405 nm) for 50 s at the same distance. Both UV- and visible light-mediated crosslinking processes were carried out under sterile conditions.

### 2.7 Rheological characterization and injectability evaluation

To evaluate the injectability of Col-MA and DNPM-MA hydrogels, shear viscosity measurements were performed prior to photocrosslinking. Col-MA and DNPM-MA precursor solution was prepared by dissolving the polymer in PBS containing 0.25 % (w/v) LAP and incubating at 37 °C until fully solubilized. Viscosity testing was conducted at 25 °C using a rotational rheometer (MCR 92, Anton Paar, Austria), with shear rates ranging from 0.1 to 100 s^−1^. All measurements were conducted in triplicate. Shear viscosity-shear rate profiles were analyzed to assess shear-thinning behavior, which reflects the flowability and suitability of the precursor for injection through narrow-gauge needles.

To characterize the viscoelastic properties of the crosslinked hydrogels, strain sweep tests were carried out using the same rheometer in strain-controlled mode. The precursor solutions were photocrosslinked under UV irradiation to form hydrogels, which were subsequently tested at 25 °C. A strain range of 1 %–1000 % was applied, and the storage modulus (G′) and loss modulus (G″) were recorded as functions of shear strain. All tests were performed in triplicate. The strain-dependent profiles of G′ and G″ were analyzed to evaluate the mechanical robustness and structural integrity of the hydrogels.

### 2.8 Morphology test of the DNPM-MA hydrogel

Col-MA hydrogels with a concentration of 5 % w/v and DNPM-MA hydrogels with different concentrations of 5 %, 8 % and 10 % w/v were prepared as described above in hexagonal star-shaped molds (with a perimeter of 28 mm and a thickness of 2.5 mm). After demolding, the hydrogel samples were photographed using a digital camera for analysis of the geometrical and shape fidelity. Subsequently, the micromorphology of the Col-MA and DNPM-MA hydrogels was observed via SEM with an accelerating voltage of 10 kV, following lyophilization and gold palladium coating procedures. The pore size was analyzed using Image J software, and the degree of porosity was measured using the gasethanol replacement method, as described in a previous study [22].

### 2.9 Mechanical and swelling properties of the DNPM-MA hydrogel

To assess the mechanical properties, a nanoindentation technique was performed to draw the strain-stress curves of 5 % w/v Col-MA and DNPM-MA hydrogels at different concentrations (5 %, 8 % and 10 % w/v). The compressive modulus of each hydrogel sample was calculated from the slope of the nanoindentation-based strain-stress curves, according to a previous study [20].

To observe the swelling properties, 5 % w/v Col-MA and different concentrations of DNPM-MA (5 %, 8 % and 10 % w/v) hydrogel precursors, along with 0.25 % w/v LAP, and then the mixtures were dissolved in PBS containing 0.5 % phenol red dye at 37 °C. Next, the hydrogel precursors were injected into circular mold cavities (6 mm in diameter, and 1 mm in thickness) and subjected to UV irradiation (365 nm, 850 mW) for 30 s. After demolding, the hydrogel samples were immersed in 0.5 ml of PBS for 24 h at room temperature and removed at defined time points. After removing the excess PBS from the surface of the hydrogel samples, the swelling weight (W_t_) of each sample was recorded. The dry weight (W_d_) was measured after freeze-drying these samples, and the swelling ratio was then calculated using the following formula: (W_t_ - W_d_)/W_d_ × 100 %.

### 2.10 Biocompatibility and biodegradation tests

To assess the cytotoxicity of DNPM-MA hydrogel, NPPCs were premixed with the DNPM-MA hydrogel precursor at a density of 1 × 10^7^ cells/mL, and injected into circular mold cavities (6 mm in diameter, and 1 mm in thickness) with subsequent UV irradiation to construct the network. Cell viability and proliferation within the hydrogels were then assessed via live/dead staining and DNA quantification, according to our previous studies [9,20]. For cytocompatibility analysis, F-actin and DAPI dual fluorescence staining was performed to visualize the cytoskeleton and nuclei of the hydrogel-encapsulated cells.

To test the degradation of DNPM-MA hydrogel, hydrogel precursors were mixed with 0.25 % w/v LAP and alkene coupled hydrogel fluorescent dyes and then dissolved in PBS at 37 °C as per the manufacturer's instructions. These precursors were injected into circular mold cavities (6 mm in diameter and 1 mm thick) and crosslinked under UV irradiation. The fluorescein-labeled hydrogels were subcutaneously implanted into nude mice, which were anesthetized for live imaging observation at defined time points. Additionally, the remaining implanted hydrogels and encapsulating subcutaneous tissues were harvested for paraffin embedding and HE staining to analyze the histocompatibility of the DNPM-MA hydrogel.

### *2.11* *Isolation and identification of rabbit* NPPCs

NP tissues utilized for NPPCs isolation were carefully separated from the lumbar IVDs of New Zealand rabbits via aseptic dissection. NPPCs were isolated as described in previous studies [[23], [24], [25]], and cultured in a complete medium consisting of DMEM/F12 supplement with 10 % fetal bovine serum and 1 % penicillin/streptomycin at 37 °C with 5 % CO_2_.

The isolated NPPCs were cultured to passage II and harvested for detection of the surface markers CD73, CD105, CD34, and HLA-DR via flow cytometry. Briefly, NPPCs were harvested, washed twice with PBS, and adjusted to a density of 1 × 10^6^ cells/mL in staining buffer (PBS containing 1 % bovine serum albumin). Cells were incubated with fluorochrome-conjugated monoclonal antibodies against CD73-APC, CD105-FITC, CD34-PE, and HLA-DR-APC-Cy7 for 30 min at 4 °C in the dark. Unstained controls were included in each batch of analysis, and the fluorescence distribution of the unstained control was used to define the negative region, with the same threshold consistently applied to all stained samples. After incubation, cells were washed twice with PBS and analyzed immediatelywithout fixation or permeabilization. All samples were measured using a CytoFLEX flow cytometer (Beckman Coulter, USA), and data were analyzed with CytExpert software (Beckman Coulter, version 2.5). All experiments were independently repeated three times.

To test trilineage differentiation potential, NPPCs were cultured in osteogenic, adipogenic, and chondrogenic differentiation media. After 3 weeks of induction per the manufacturer's instructions, the adherent cells/suspended microtissues were fixed and stained with Alizarin Red, Oil Red, Alcian blue, and Safranin O to evaluate differentiation outcomes. The stained areas were observed and scanned under an optical microscope.

### 2.12 Cultivation of NP-MTs using a hydrostatic bioreactor

A customized unit with multiple microwells at the bottom was utilized for culturing NPPC spheroids, which was suitable for the hydrostatic bioreactor's tissue culturing chamber. According to the manufacturer's instructions, an NPPCs suspension at a concentration of 20 million cells/ml was prepared, and 0.25 ml of this suspension was injected into a customized unit produced by Shenzhen Lingxi Technology Co., Ltd, which could generate approximately 500 cellular spheroids for the subsequent cultivation of microtissues. A gradient of low-magnitude hydrostatic pressure was then applied to the chambers by the bioreactor for 4 weeks of in vitro culture.

To assess cell viability, Live/Dead staining was performed following the manufacturer's instructions. In brief, the samples were incubated with 1 ml of working fluid at 37 °C for 30 min in the dark. Afterward, the samples were observed and imaged using a laser scanning confocal microscope. Additionally, the proliferation curves of the cells within the NP-MTs were measured using a CCK-8 assay, as described in our previous study. NP-MTs were harvested at defined time points and seeded into a 96-well plate. Subsequently, 10 μl of CCK-8 working fluid was added to 90 μl of culture media in each well and incubated for another 2 h at 37 °C. Proliferation curves were generated based on the OD values of each well.

### 2.13 Analysis of histological characteristics and components of NP-MTs

To observe the histological characteristics and matrix distribution, NP-MT samples were harvested at defined time points and embedded in paraffin. After being sliced into sections, the samples were stained with HE, Alcian blue, and Safranin O dyes following the manufacturer's instructions. Additionally, the expression of GAG and Col II in the NP-MT samples was quantified using ELISA kits, also according to the manufacturer's instructions.

### 2.14 Assessments of TE-NP-based NP regeneration in vitro

Gradient concentrations of NP-MTs (1, 2, 3, and 4 × 10^3^ spheroids/ml) were mixed with the 10 % w/v DNPM-MA precursor, injected into the hexagonal star-shaped cavities (perimeter of 28 mm, and thickness of 2.5 mm) of a silicone mold, and then crosslinked under UV irradiation. After demolding, the TE-NP samples with varying microtissue seeding densities were successfully constructed and photographed for geometrical and shape fidelity analysis. Next, nanoindentation was used to generate strain-stress curves, and the compressive modulus of each sample was calculated from the slopes of these curves, as described in a previous study [20].

Based on the compressive modulus data, NP-MTs encapsulated by Col-MA/DNPM-MA at a concentration of 4 × 10^3^ spheroids/ml were identified as the optimal parameter for TE-NP construction. Two types of TE-NPs were then transferred into hydrostatic bioreactor chambers for 4 weeks of in vitro culture. Samples were harvested at defined time points for HE and Alcian blue staining. Additionally, the contents of DNA, GAG, and Col II in the regenerated tissues derived from the TE-NP samples were quantified using ELISA kits according to the manufacturer's instructions.

### 2.15 Transcriptomic analysis

To determine the potential mechanisms involved in the regeneration process induced by the DNPM-MA hydrogel, RNA sequencing was performed to analyze differences in the transcriptome between in vitro cultured NP-MTs@Col-MA and NP-MTs@DNPM-MA. After 2 weeks of culture in the bioreactor, the two types of TE-NP samples were harvested for total RNA extraction, high-throughput sequencing, and corresponding RNA library construction (Majorbio Biotech Co., Ltd., China). Sequencing data was analyzed using DESeq software, and significant differentially expressed genes (DEGs) between the two groups were measured using the transcripts per million reads method (log fold change ≥1, p-adjusted <0.05). Additionally, in-depth bioinformatics analyses, including GO and Kyoto Encyclopedia of Genes and Genomes (KEGG) analyses, were employed to identify GO terms and metabolic pathways in which DEGs were significantly enriched, with a Bonferroni-corrected P value ≤ 0.05 compared to the whole-transcriptome background.

### 2.16 Western blotting assessment

Based on the RNA sequencing data, Western blotting was performed to assess the changes in protein expression between the two groups of tissues. Briefly, total proteins were extracted using a protein extraction kit. Specific primary antibodies against IL1β, p-p38, ACAN, Col II, ASK1, MMP9 and MMP13 were utilized to detect protein expression levels. Additionally, a SA-β-gal assay was utilized to detect the degree of senescence of the cells in Col-MA/DNPM-MA hydrogel-encapsulated NP-MTs.

### 2.17 Inflammaging-associated pathway inhibition assay using p38 MAPK inhibitor

The NP-MTs were encapsulated in either Col-MA or DNPM-MA hydrogels at a concentration of 4 × 10^3^ spheroids/ml, which was previously identified as the optimal parameter for TE-NP construction. The resulting TE-NPs were transferred into hydrostatic bioreactor chambers and pre-cultured for 3 days to allow initial gel stabilization and matrix integration. Subsequently, the chambers were supplemented with SB203580, a selective p38 MAPK inhibitor, at a final concentration of 10 μM, based on prior studies involving NPPCs [26,27], and cultured for an additional 7 days under hydrostatic conditions.

At the endpoint, samples were harvested for multiple analyses. Cellular senescence was assessed by SA-β-gal staining, while intracellular ROS levels were evaluated using a DCFH-DA fluorescence assay. For ROS detection, cells were collected, washed twice with PBS, and incubated with 5 μM DCFH-DA in serum-free medium at 37 °C for 30 min in the dark. After incubation, cells were washed twice with PBS to remove excess probe, and green fluorescence was immediately detected using the FITC channel (excitation: 495 nm, emission: 530 nm). In addition, immunofluorescence (IF) staining and Western blotting were performed to analyze the expression of key signaling and ECM remodeling markers, including p-p38, Nox4, IL-1β, ASK1, collagen II, and MMP13.

### 2.18 Histological staining

Histological immunohistochemistry (IHC) and IF staining were performed to evaluate the expression of ECM metabolism and inflammaging-associated biomarkers in the regenerated tissues derived from the two types of TE-NP. Briefly, the sections were deparaffinized, hydrated, and then unmasked with trypsin/EDTA. For IHC staining, half of the sections were incubated with primary antibodies against IL-1β and p21. Afterward, the sections were incubated with an IgG-HRP secondary antibody, counterstained with Harris's hematoxylin, and imaged under an optical microscope. For IF staining, the other half of the sections were incubated with primary antibodies against Col II and MMP13. The sections were then incubated with a fluorescent dye-conjugated secondary antibody, labeled with DAPI, and scanned under a laser scanning confocal microscope (Leica, Germany). The average optical density (AOD) values of five randomly selected visual fields (per immunohistochemically or immunofluorescently stained section) were analyzed using ImageJ software.

### 2.19 Assessments of TE-NP-based NP regeneration in vivo

All procedures were approved by the Institutional Animal Care and Use Committee (IACUC) of Chongqing Medical University (IACUC-CQMU-2023-0148). Male New Zealand rabbits at skeletal maturity, obtained from the CQMU Laboratory Animal Center, were randomly assigned to four groups. The control group did not undergo any surgical procedures on the IVDs. The rabbit in the resection group underwent partial NP excision from their IVDs. In the NP-MTs@Col-MA implantation group, partial NP excision followed by in situ implantation of NP-MTs@Col-MA was performed. The NP-MTs@DNPM-MA implantation group also underwent partial NP excision, followed by in situ implantation of NP-MTs@DNPM-MA. All surgeries were performed under inhalation anesthesia.

A radiological evaluation using MRI revealed the hydration status of the NP tissue, and the degeneration scores were then evaluated in the T2-weighted images according to the Pfirrmann classification system. After euthanasia, the rabbits’ IVDs were harvested and cut into sections. The sections were then subjected to HE, Alcian blue, Safranine O-fast green, Col I and Col II IHC staining, as described in our previous study. The histological degree of IVD degeneration was evaluated using a histopathology scoring system. In addition, for biomechanical analysis, freshly isolated NP tissues were subjected to nanoindentation testing. Stress-strain curves were generated, and the compressive modulus was calculated from the linear region of each curve to evaluate the mechanical integrity of the regenerated NP tissue.

### 2.20 Statistical analysis

All experiments were repeated at least three times, and data are presented as means ± standard deviation (SD). Statistical analyses were performed using GraphPad Prism 9.0 software. Prior to statistical testing, all datasets were evaluated for normality using the Shapiro–Wilk test. For comparisons between two groups, unpaired two-tailed Student's t-tests were used when the data followed a normal distribution. For datasets that did not meet this assumption, non-parametric alternatives such as the Mann-Whitney *U* test were applied. For comparisons among multiple groups, one-way ANOVA or the Kruskal-Wallis test was used depending on the distribution characteristics. Statistical significance was defined as follows: ^#^p ≥ 0.05, ∗p < 0.05, ∗∗p < 0.01, ∗∗∗p < 0.001, and ∗∗∗∗p < 0.0001. Schematic illustrations were created using BioRender.com (Confirmation of publication and licensing rights certificate can be provided upon request).

## 3 Results

### 3.1 Preparation and characterization of DNPM-MA hydrogels

**Fig. 2:**
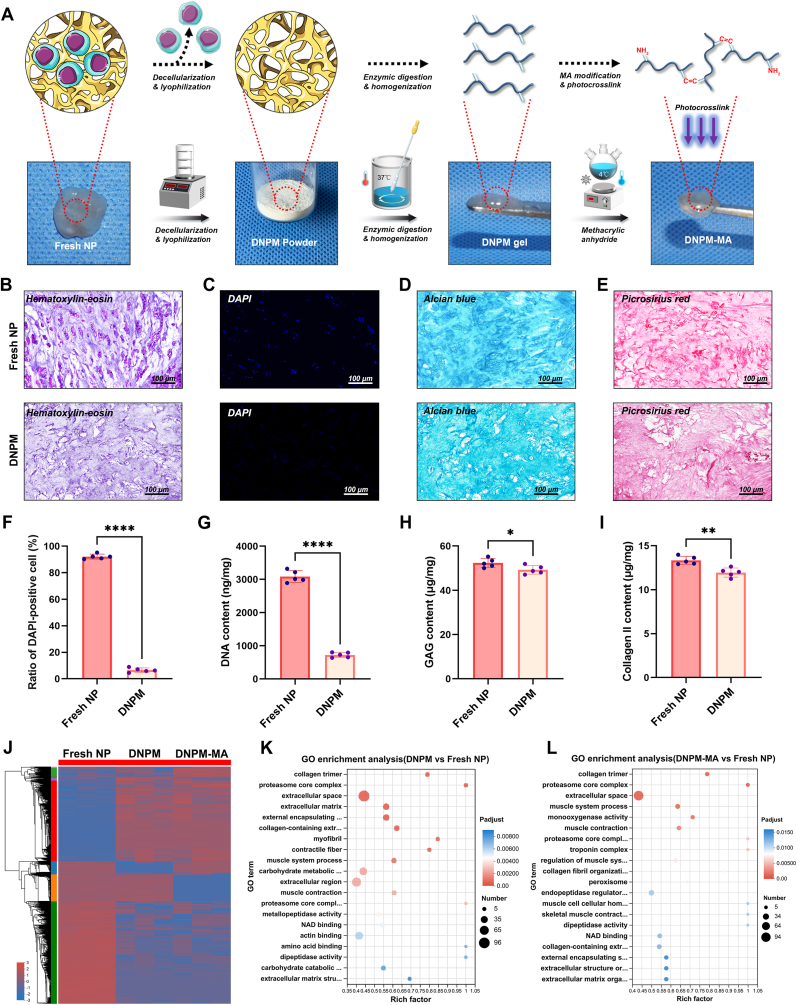
Preparation and composition analysis of the DNPM-MA hydrogel. (A) Schematic workflow of the fabrication of photocrosslinkable DNPM-MA hydrogel. (B–E) Representative histological images of fresh and decellularized NP tissues after HE (B), DAPI (C), Alcian blue (D), and Picrosirius red (E) staining. (F–I) Statistical analysis of the DAPI-positive cell ratio (F) and DNA (G), GAG (H), and collagen II (I) contents of the fresh and decellularized NP tissues. (J) Heatmap of the TMT-based quantitative proteomic analysis revealing the DEPs among fresh NP, DNPM, and DNPM-MA. (K) GO analysis of the DEPs between the fresh NP and DNPM groups. (L) GO analysis of the DEPs between the fresh NP and DNPM-MA groups. ∗p < 0.05, ∗∗p < 0.01, and ∗∗∗∗p < 0.0001 were considered to indicate statistical significance. (For interpretation of the references to colour in this figure legend, the reader is referred to the Web version of this article.)

To date, a variety of DNPM-based hydrogels have been developed and applied in the construction of engineered analogs for treating DDD, yielding positive regenerative outcomes [28,29]. However, current DNPM-based hydrogels still face certain limitations, such as a relatively weak mechanical strength and potential cytotoxicity [30]. Hence, in the present study, a novel photocrosslinkable DNPM-based hydrogel was fabricated with the protocol as follow: a. preparation of powder-like DNPM by decellularization and lyophilization processes; b. gelation of DNPM powders using a modified “Freytes method” [31], which involved a combination of enzymatic digestion and homogenization procedures; and c. methacrylation of DNPM to produce DNPM-MA, where a common methacrylation reaction was utilized to synthesize photocrosslinkable hydrogels, similar to methacrylate-modified gelatin (Gel-MA) [32], methacrylate-modified hyaluronic acid (HA-MA) [21], and other methacrylate-modified decellularized cartilage-based hydrogels [33,34] (Fig. 2A). Porcine NP tissue was used as a source material to prepare DNPM gels, which were subsequently fabricated into the DNPM-MA hydrogels. Chemical (using the Triton method) and biological (using deoxyribonuclease and ribonuclease) decellularization were combined to produce DNPM, as described in our previous study [20]. The results of HE staining, DAPI staining, and quantification of the DNA content in the fresh and decellularized NP samples revealed that this strategy achieved satisfactory decellularization outcomes (Fig. 2B–C, and Fig. 2F–G). The contents of GAG and Col II, key components of NP matrix, were assessed using Alcian blue staining, Picrosirius Red staining, and quantitative ELISA. The results indicated that the GAG and Col II contents were slightly lower in the decellularized NP samples than in the fresh NP tissue samples (Fig. 2D–E, and Fig. 2H–I). Furthermore, quantitative proteomics was applied to identify and compare DEPs mong fresh NP, DNPM, and DNPM-MA (Fig. 2J). The GO enrichment analysis suggested that the major DEPs between both the fresh NP group and the DNPM group, and between the fresh NP group and the DNPM-MA group were related to similar biological processes, involving collagen trimers, proteosome core complexes, and the extracellular space (Fig. 2K–L). These findings suggested that the DNPM-MA hydrogel substantially preserved the functional constituents of the NP matrix and was capable of performing analogous biological functions.

**Fig. 3:**
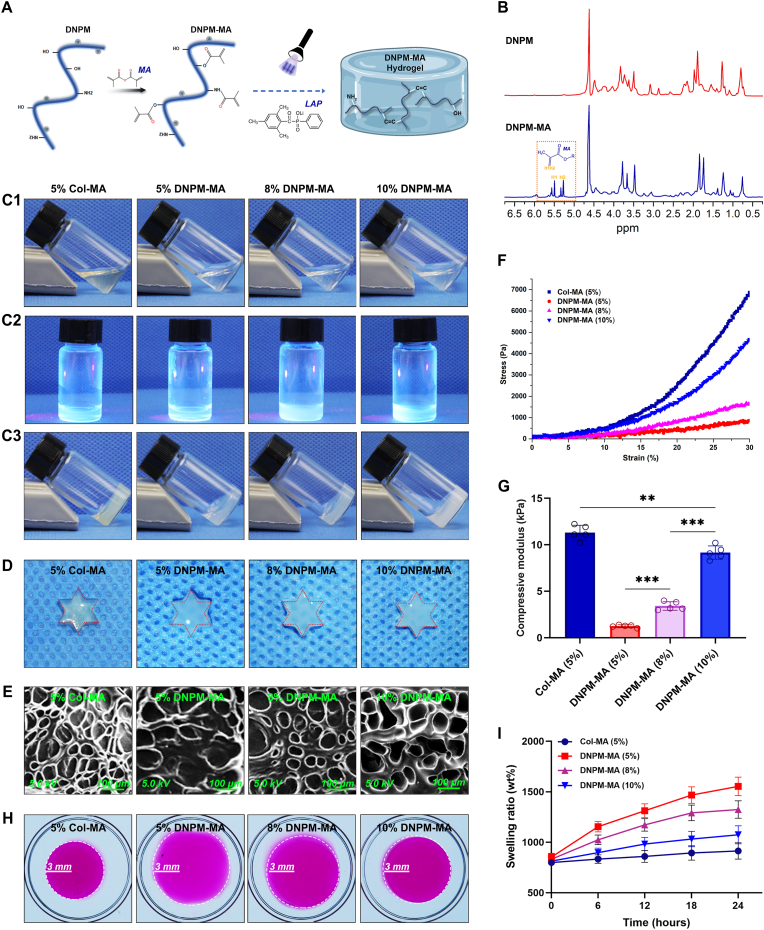
Characterization of the Col-MA and DNPM-MA hydrogels. (A) Schematic representation of the photocrosslinking reaction mechanism of the DNPM-MA hydrogel. (B) ^1^H NMR spectra of DNPM and DNPM-MA. (C1-3) Photographs of the sol-to-gel transition of the 5 % Col-MA and 5 %, 8 %, and 10 % DNPM-MA hydrogels under UV light (365 nm, 20 mW cm^−2^). (D) Gross view and shape analysis of the crosslinked 5 % Col-MA and 5 %, 8 %, and 10 % DNPM-MA hydrogels. (E) Representative SEM images of the crosslinked 5 % Col-MA and 5 %, 8 %, and 10 % DNPM-MA hydrogels. (F) Stress-strain curves of the crosslinked 5 % Col-MA and 5 %, 8 %, and 10 % DNPM-MA hydrogels. (G) Compressive modulus of the crosslinked 5 % Col-MA and 5 %, 8 %, and 10 % DNPM-MA hydrogels. (H–I) Analysis of the swelling capability of the crosslinked 5 % Col-MA and 5 %, 8 %, and 10 % DNPM-MA hydrogels. ∗∗p < 0.01 and ∗∗∗p < 0.001 were considered to indicate statistical significance.

DNPM-MA was synthetized based on a methacrylation reaction (Fig. 3A). To verify the successful incorporation of methacrylate groups, FTIR spectroscopy was performed. The presence of characteristic peaks corresponding to C=C stretching vibrations confirmed the successful introduction of methacrylate moieties into the modified hydrogel. The FTIR data are presented in the supplementary material (Fig. S1A–B). Furthermore, ^1^H NMR spectroscopy of DNPM-MA revealed signal peaks at 5.2–5.8 ppm, indicating the successful grafting of methacrylate groups to the amino groups of DNPM (Fig. 3B). To quantify the degree of substitution, the integrated area of the lysine methylene protons (δ = 2.9–3.1 ppm), corresponding to the primary amine groups, was compared between unmodified DNPM and DNPM-MA. The methacrylation substitution degree (%) was calculated using the following equation: (1-A_DNPM-MA_/A_DNPM_) × 100 %, where A_DNPM-MA_ and A_DNPM_ represent the integrated peak areas of lysine methylene protons in DNPM-MA and unmodified DNPM, respectively. Based on this analysis, the degree of methacrylation of DNPM-MA was determined to be 49.58 ± 1.25 %. Considering that the NP matrix is mainly composed of Col II, we modified porcine-derived Col II through a methacrylation reaction to prepare a Col-MA hydrogel [35], which served as a benchmark control. To evaluate the crosslinking behavior, precursor solutions of DNPM-MA and Col-MA containing 0.15 % (w/v) LAP were subjected to photocrosslinking. Upon exposure to UV light (365 nm) for 30 s or alternatively to visible light (405 nm) for 50 s at the same distance, both hydrogels rapidly underwent gelation and formed stable three-dimensional (3D) networks (Fig. 3C1-3).

Subsequently, we investigated the shape maintenance capacity of these hydrogels by preforming them in hexagonal star-shaped molds. Apart from the 5 % DNPM-MA hydrogel, the geometries of the 5 % Col-MA and the 8 % and 10 % DNPM-MA hydrogels exhibited a relatively high degree of shape fidelity after demolding (Fig. 3D, Fig. S2A–B). As shown in Fig. 3E, the SEM analysis of the crosslinked hydrogel samples revealed that the pore size decreased as the concentration of the DNPM-MA hydrogel increased. Notably, both the 5 % Col-MA and the 10 % DNPM-MA hydrogels displayed relatively uniform porous microstructures with comparable pore dimensions and porosities (Fig. 3E, Fig. S2C–D). A mechanical analysis of the crosslinked hydrogel samples indicated that the compressive moduli of all DNPM-MA hydrogels were lower than those of the Col-MA group (Fig. 3F–G, Fig. S1C–D). Nevertheless, a significant increase in the mechanical properties was observed with increasing DNPM-MA concentration. Specifically, the 10 % DNPM-MA hydrogel exhibited a compressive modulus adequate to meet the mechanical property requirements for constructing TE-NP (Fig. 3F–G), according to previous studies [5,19]. Furthermore, the swelling capability of the DNPM-MA hydrogels was greater than that of the Col-MA hydrogels and decreased with increasing DNPM-MA concentration (Fig. 3H–I). The swelling capacity of the 5 % Col-MA and 10 % DNPM-MA hydrogels was suitable for the fabrication of injectable TE-NP, as they maintained an appropriate volume after crosslinking, preventing overexpansion of the intradiscal cavity (Fig. 3H–I). Rheological strain sweep tests were conducted to evaluate and compare the gelation behavior and mechanical robustness of 10 % DNPM-MA and 5 % Col-MA hydrogels. As shown in the Supplementary Material (Fig. S1C–D), both hydrogels exhibited typical viscoelastic gel-like behavior, with the storage modulus (G′) consistently exceeding the loss modulus (G″) across a wide strain range, indicating successful network formation. Notably, the overall G′ values of 10 % DNPM-MA and 5 % Col-MA were comparable, suggesting similar crosslinking efficiency and stiffness at the tested concentrations. However, 10 % DNPM-MA showed an earlier onset of modulus decline at higher strain, indicating relatively lower structural resilience under mechanical deformation compared to Col-MA.

### 3.2 Evaluation of the injectability, biocompatibility, and biodegradation of the hydrogels

**Fig. 4:**
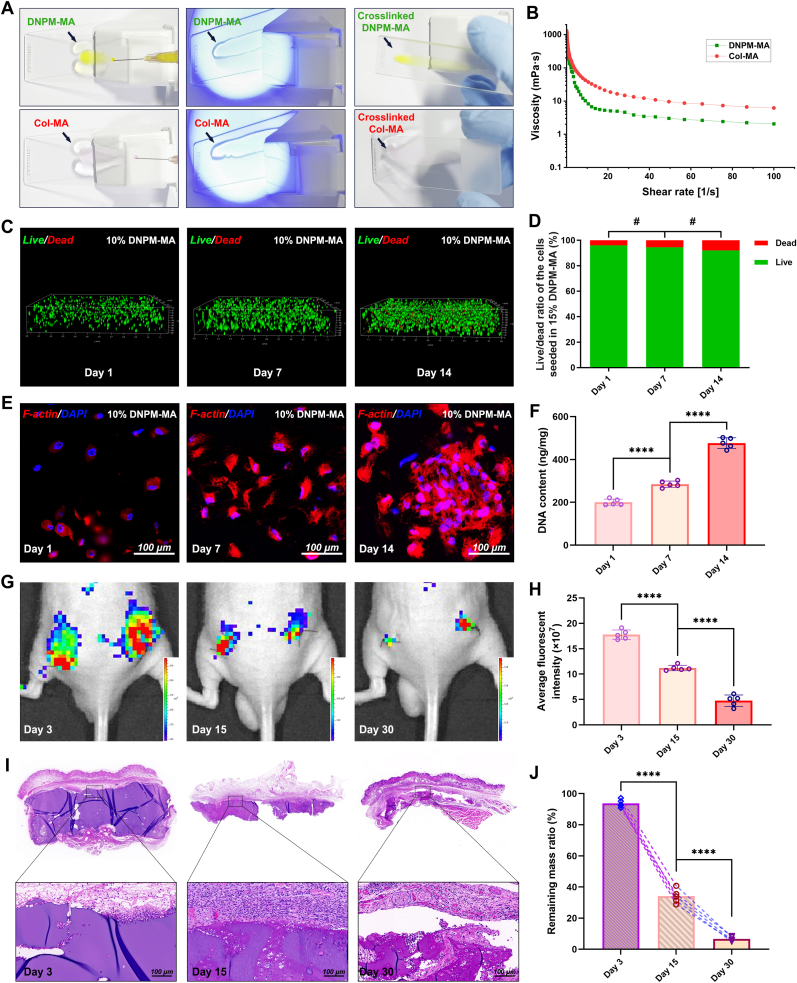
Injectability and biocompatibility assessments of the hydrogels. (A) Observation of injectability for Col-MA and DNPM-MA hydrogel precursor solutions. (B) Viscosity-shear rate profiles of Col-MA and DNPM-MA hydrogel precursor solutions. (C) The viability of NPPCs seeded in DNPM-MA hydrogels was assessed by live/dead staining on Days 1, 7, and 14. (D) Statistical analysis of the cell live/dead ratio of the NPPCs seeded in the DNPM-MA hydrogel on Days 1, 7, and 14. (E) Cytoskeleton of the NPPCs seeded in the DNPM-MA hydrogel was assessed by F-actin/DAPI staining on Days 1, 7, and 14. (F) The viability of the NPPCs seeded in the DNPM-MA hydrogel was assessed by quantifying DNA on Days 1, 7, and 14. (G–H) The in vivo degradability of the DNPM-MA hydrogel was assessed by fluorescent living imaging (G), and the average flurescent intensity of each sample was statistically analyzed (H). (I–J) The HE staining (I), and determination (J) of the remaining mass. **^#^**p > 0.05 was considered not statistically significant. ∗∗∗∗p < 0.0001 was considered to indicate statistical significance.

Both Col-MA and DNPM-MA hydrogels demonstrated excellent injectability, as shown in Fig. 4A and Supplementary Video. Shear viscosity measurements revealed distinct differences in the injectability of DNPM-MA and Col-MA precursor solutions (Fig. 4B). As shown in Fig. 4B, both hydrogels exhibited typical shear-thinning behavior, with viscosity decreasing as shear rate increased, indicating favorable flow characteristics for injection. Notably, DNPM-MA consistently demonstrated lower viscosity than Col-MA across the entire shear rate range (0.1–100 s^−1^), suggesting superior injectability and lower resistance during extrusion through narrow-gauge needles. The aforementioned results indicated that the 10 % DNPM-MA hydrogel displayed proper crosslinking efficiency, microstructural features, and a balance of mechanical strength and swelling properties, and was suitable for the construction of TE-NPs. Hence, hydrogels composed of 10 % DNPM-MA were further selected for evaluations of cytocompatibility, histocompatibility, and biodegradation via live/dead staining, DNA quantification, F-actin fluorescence staining, and in vivo degradation tests. Live/dead staining revealed that NPPCs encapsulated in 10 % DNPM-MA hydrogel maintained adequate viability, with minimal cell death (less than 10 %) observed during a one-week period of in vitro culture (Fig. 4C–D). The findings from F-actin staining illustrated that the host cells could spread and maintain their normal cytoskeleton within the 10 % DNPM-MA hydrogel during a one-week period of in vitro culture (Fig. 4E), confirming their favorable cytocompatibility. The results of the quantitative DNA analysis also confirmed that cells encapsulated in the 10 % DNPM-MA hydrogel exhibited robust cell proliferation (Fig. 4F).

Moreover, living imaging observation of the subcutaneously implanted DNPM-MA hydrogels labeled with fluorescein elucidated the temporal in vivo degradation dynamics of the hydrogels (Fig. 4G). Specifically, by Day 15 postimplantation, the fluorescence emitted from DNPM-MA was markedly diminished, indicating substantial degradation of the material. This trend became even more evident at the 30-day mark, with the fluorescence signal becoming almost undetectable, suggesting that the hydrogels had undergone near-complete degradation (Fig. 4H). Additionally, the histological evidence provided by HE staining of the remaining hydrogels subcutaneously implanted into nude mice was consistent with the living imaging observations (Fig. 4I). The HE staining results showed that the subcutaneously implanted DNPM-MA hydrogels underwent gradual degradation over time, with no notable rejection response detected, confirming the excellent biodegradability and biocompatibility of the DNPM-MA hydrogels (Fig. 4J).

### 3.3 Cultivation of NP-MTs using a hydrostatic pressure bioreactor

**Fig. 5:**
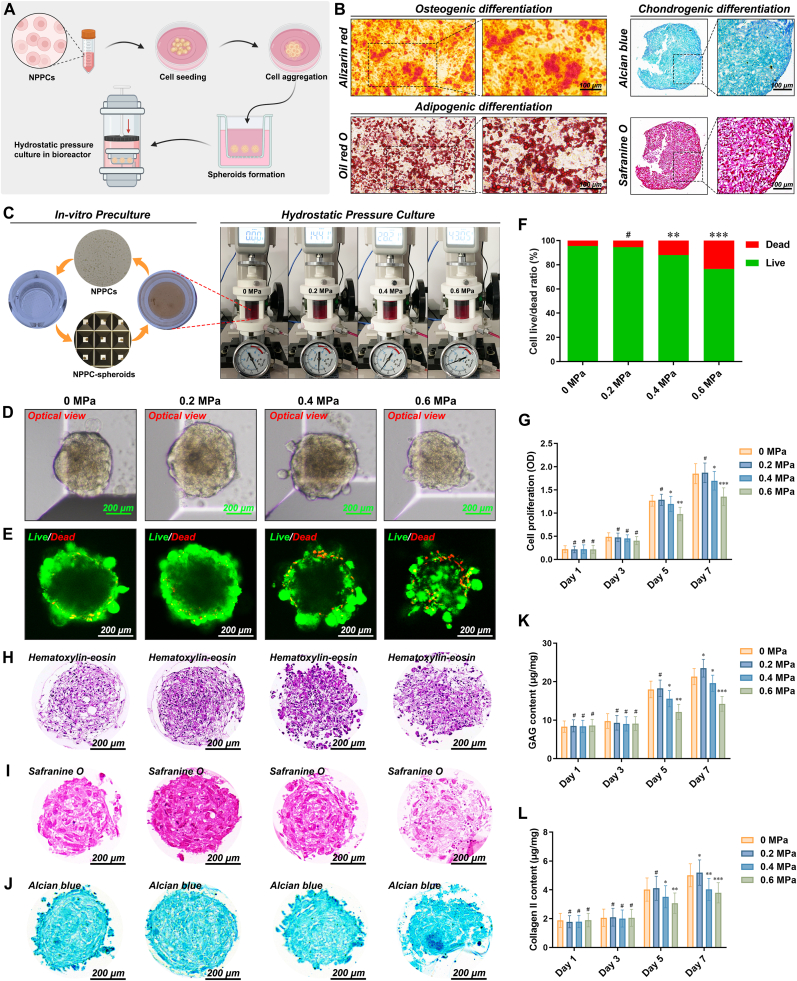
Hydrostatic bioreactor-based cultivation of NP-MTs in vitro. (A) Schematic illustration of the NP-MT construction and cultivation procedures using our self-developed hydrostatic bioreactor. (B) Representative images of Alizarin red, Oil red O, Alcian blue, and Safranine O staining after the osteogenic, adipogenic, and chondrogenic differentiation of NPPCs. (C) Photographs of the NPPC spheroidizing culture insert, tissue cultivation chambers, and loading application devices of the hydrostatic bioreactor. (D) Optical microscopy observations of NP-MTs cultivated under different degrees of hydrostatic pressure. (E) The viability of the NPPCs in NP-MTs cultivated under different degrees of hydrostatic pressure was assessed using live/dead staining. (F) Statistical analysis of the percentages of live/dead NPPCs in NP-MTs cultivated under different degrees of hydrostatic pressure. (G) Changes in the viability of NPPCs in NP-MTs cultivated under different degrees of hydrostatic pressure were determined using a CCK-8 assay on Days 1, 3, 5, and 7, and cell proliferation was determined by constructing optical density (OD) curves. (H–J) Representative histological images of HE (H), Safranine O (I), and Alcian blue (J) staining of NP-MTs cultivated under different degrees of hydrostatic pressure. (K–L) Quantification of the GAG (K) and collagen II (L) contents of the NPPCs in NP-MTs cultivated under different degrees of hydrostatic pressure was performed using ELISA kits on Days 1, 3, 5, and 7. **^#^**p > 0.05 was considered not statistically significant. ∗p < 0.05, ∗∗p < 0.01, and ∗∗∗p < 0.001 were considered to indicate statistical significance. (For interpretation of the references to colour in this figure legend, the reader is referred to the Web version of this article.)

Previous investigations have shown that cell spheroids could be utilized as viable seed units for the construction of engineered NP analogs, exhibiting promising outcomes in tissue regeneration [9,36]. Building on this foundation, we cultivated NPC-based spheroids under bionic hydrostatic pressure conditions to enhance the generation of matrix surroundings, thereby promoting the development of microtissues (Fig. 5A). Additionally, staining with Alizarin red, Oil Red O, Alcian blue, and Safranine O demonstrated that NPPCs possessed multipotent trilineage differentiation capabilities, thereby corroborating the potential of NPPCs for mediating NP regeneration (Fig. 5B). Recent high-impact studies have identified a heterogeneous population within the adult NP niche, including residual notochordal cells, chondrocyte-like cells, and a subset of regenerative NPPCs that exhibited stem/progenitor phenotypes—commonly referred to as nucleus pulposus-derived stem/progenitor like cells (NPPCs/NPSCs) [25,37]. In line with this updated understanding, we harvested NPPCs at passage II and evaluated their phenotype through biomarker analysis and trilineage differentiation assays. Flow cytometry revealed positive expression of CD73 and CD105, and negative expression of CD34 and HLA-DR (Fig. S3), indicating that these cells met the criteria for stem/progenitor-like cells [23]. As illustrated in the schematic photographs of the NP-MT formation procedure (Fig. 5C), the NPPC suspension was seeded into a customized unit with multiple microwells at the bottom for 3D spheroidizing culture. These spheroids were then transferred into the chambers of a hydrostatic bioreactor (Fig. S4) for 4 weeks of biomimetic culture under varying low-magnitude hydrostatic pressures (Fig. 5C, Fig. S5A–C). Optical microscopy observations revealed that the diameter of all groups of NP-MTs plateaued at approximately 300 μm (Fig. 5D). Notably, the NP-MTs cultivated under 0.2 MPa of hydrostatic pressure displayed the largest diameter and a more spherical morphology (Fig. 5D). Furthermore, the viability of the NPPCs within the NP-MTs was assessed using live/dead staining. Notably, living NPPCs constituted approximately 90 % of the total cell number in the NP-MTs cultivated under a hydrostatic pressure of 0.2 MPa (Fig. 5E–F). However, the application of hydrostatic pressures exceeding 0.4 MPa led to a substantial reduction in cell viability within the NP-MTs (Fig. 5E–F). Variations in the viability of NP-MTs cultured under different magnitudes of hydrostatic pressure were assessed using the CCK-8 assay on Days 1, 3, 5, and 7. According to the cell proliferation curves, no marked changes in cell viability were found in any group of NP-MTs (Fig. 5G). However, after 5 days of culture, NPPCs in NP-MTs exposed to hydrostatic pressures exceeding 0.4 MPa exhibited a noticeable decrease in cell viability, consistent with the results of live/dead staining (Fig. 5G).

Histological staining and quantification of ECM components were further performed to evaluate the microtissue formation process. After in vitro cultivation for four weeks under optimized conditions, NPPC-derived spheroids were capable of developing into NP-MTs with abundant deposition of hydrophilic ECM components (Fig. S5 A-C). Specifically, after 4 weeks of in vitro cultivation, the NP-MTs cultured under 0.2 MPa exhibited a fine histological structure (Fig. 5H) and the most abundant ECM deposition among all the groups of microtissues cultured under different hydrostatic pressures (Fig. 5I–J). Nevertheless, when cultured under 0.4 MPa or 0.6 MPa hydrostatic pressure for 4 weeks, the matrix structure was obviously disrupted, and the staining intensity decreased. Specifically, after a four-week period of in vitro cultivation, the NP-MTs subjected to 0.2 MPa displayed a superior histological structure (Fig. 5H) and the most substantial ECM deposition (Fig. 5I–J). Nevertheless, when cultured under hydrostatic pressures of either 0.4 MPa or 0.6 MPa for four weeks, a noticeable disruption in the matrix structure was observed (Fig. 5H), accompanied by a decrease in the intensity of Safranine O (Fig. 5I) and Alcian blue staining (Fig. 5J). Additionally, ELISA quantification of the major matrix components (GAG and Col II) was performed for the NP-MTs cultivated under different degrees of hydrostatic pressure on Days 1, 3, 5, and 7. The findings revealed that the secretion of both GAG and Col II decreased significantly in the groups treated with 0.4 MPa and 0.6 MPa hydrostatic pressure after a five-day culture period (Fig. 5K–L). Notably, the synthesis of GAG and Col II was markedly enhanced by the application of 0.2 MPa of hydrostatic pressure for 7 days, indicating that 0.2 MPa of hydrostatic pressure positively stimulated the accumulation of the NP matrix (Fig. 5K–L).

### 3.4 DNPM-MA enhanced the regenerative efficiency of NP-MTs in vitro

**Fig. 6:**
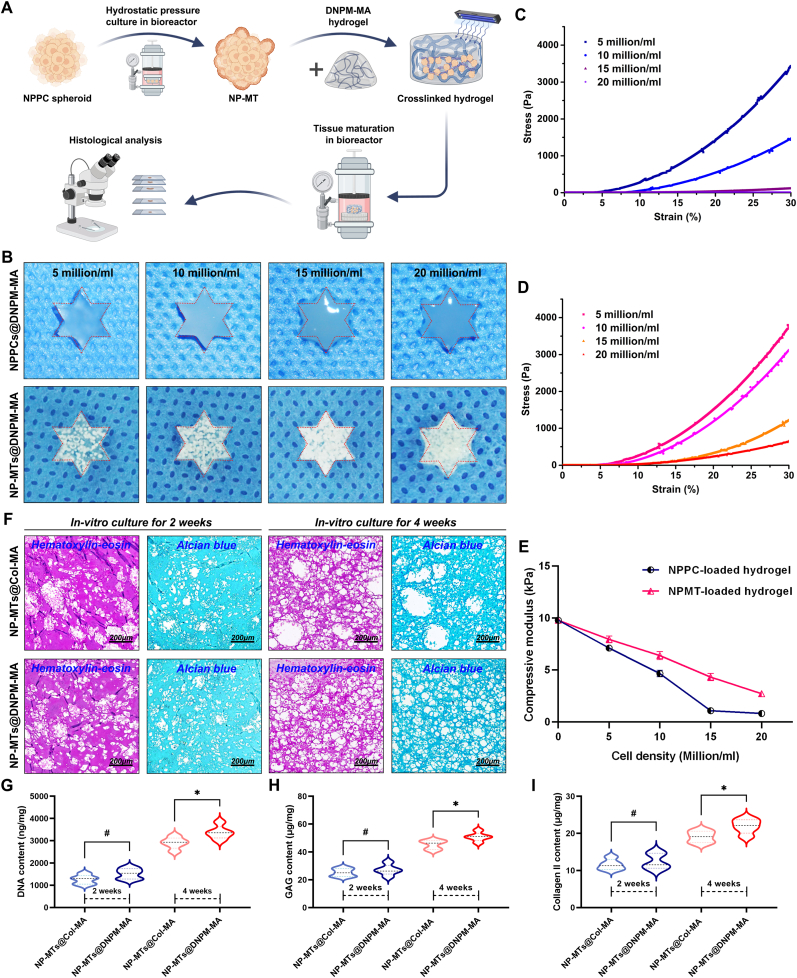
Hydrostatic bioreactor-based cultivation of TE-NPs in vitro. (A) Schematic illustration of the NP-MT-composed TE-NP cultivation procedures in vitro using our self-developed hydrostatic bioreactor. (B) Gross view and shape analysis of the NPPCs/NP-MTs-loaded DNPM-MA hydrogels with different cell/microtissue seeding densities. (C) Stress-strain curves of the NPPCs-loaded DNPM-MA hydrogels with different cell seeding densities. (D) Stress-strain curves of the NP-MTs-loaded DNPM-MA hydrogels with different microtissue seeding densities. (E) Compressive modulus of the NPPCs/NP-MTs-loaded DNPM-MA hydrogels with different microtissue seeding densities. (F) Representative histological images of HE and Alcian blue staining of the Col-MA/DNPM-MA-constructed TE-NP cultivated in a hydrostatic bioreactor for 2 and 4 weeks. (G–I) Quantification of the DNA (G), GAG (H) and collagen II (I) content of the NPPCs in NP-MTs cultivated in a hydrostatic bioreactor for 2 and 4 weeks. **^#^**p > 0.05 was considered not statistically significant. ∗p < 0.05 was considered to indicate statistical significance. (For interpretation of the references to colour in this figure legend, the reader is referred to the Web version of this article.)

Two types of TE-NP constructed from Col-MA hydrogel-encapsulated NP-MTs (NP-MTs@Col-MA) and DNPM-MA hydrogel-encapsulated NP-MTs (NP-MTs@DNPM-MA) were cultured in a hydrostatic bioreactor for 4 weeks to verify the efficiency of the DNPM-MA and Col-MA hydrogels in promoting NP regeneration (Fig. 6A). The shape retention ability and mechanical properties of the DNPM-MA hydrogel were evaluated after the encapsulation of cells or microtissues. First, the NPPC suspensions were prepared, and the same concentrations of single-cell suspensions were then inoculated into standard flasks for monolayer culture and customized microwell units for spheroidizing culture. According to the manufacturer's instructions, a single-cell spheroid contained approximately 5 × 10^3^ cells. After one week of in vitro culture, different concentrations of NPPC suspensions (5, 10, 15, and 20 million/ml) and NP-MTs (1, 2, 3, and 4 × 10^3^ spheroids/ml) were mixed with the DNPM-MA precursor and injected into hexagonal star-shaped molds. After 15 s of exposure to UV irradiation, the hydrogel-encapsulated NPPCs/NP-MTs were completely crosslinked and demolded from the molds. The geometrical analysis of the hydrogel-encapsulated NPPCs/NP-MTs indicated that the encapsulation of NP-MTs was beneficial for preserving the shape fidelity of the hydrogels (Fig. 6B). Additionally, the stress-strain curves depicted in Fig. 6C for NPPCs and Fig. 6D for NP-MTs encapsulated in DNPM-MA hydrogels showed that the encapsulation of NPPCs resulted in a significant reduction in the compressive modulus (Fig. 6E). The aforementioned investigations substantiated that the encapsulation of NP-MTs within the DNPM-MA hydrogel achieved the highest seed cell loading efficiency while causing minimal deterioration of the mechanical properties.

The elastic modulus parameters of healthy human NP tissues display a wide range of variability, ranging from 0.3 to 5 kPa [38]. Moreover, studies on the elastic modulus parameters of the NP in nonupright walking animals have also revealed significant discrepancies, with values varying between 0.345 and 0.8 kPa [38,39]. In particular, a previous study using atomic force microscopy (AFM) have reported an apparent elastic modulus of the normal rabbit NP of approximately 2.0 kPa, reflecting microscale stiffness [40]. Considering the parameters reported by prior studies and the present research, a density of 4 × 10^3^ NP-MTs per milliliter of hydrogel was determined to be the optimal parameter for TE-NP construction, with the aim of maximizing the cell seeding concentration while maintaining the shape integrity and mechanical properties of the TE-NP. After being cultivated in a hydrostatic bioreactor, two types of TE-NP, NP-MTs@Col-MA and NP-MTs@DNPM-MA, were harvested for histological examination and the quantification of ECM components. The images of HE and Alcian blue staining revealed that the nascent ECM morphology of the regenerated tissues derived from NP-MTs@DNPM-MA was more regular and denser than that of the regenerated tissues derived from NP-MTs@Col-MA during a four-week period of in vitro culture using the hydrostatic bioreactor (Fig. S6A-B, Fig. 6F). Notably, more voids were observed in the regenerated tissues derived from NP-MTs@Col-MA after 4 weeks of culture (Fig. S6A-B, Fig. 6F). Furthermore, the DNA content of NP-MTs@DNPM-MA was significantly higher than that of NP-MTs@Col-MA after 4 weeks of in vitro culture (Fig. 6G), indicating that cell proliferation of the NP-MTs was promoted by the encapsulation of the DNPM-MA hydrogel. A quantitative analysis of the NP matrix components revealed that the DNPM-MA hydrogel-encapsulated NP-MTs secreted higher amounts of GAG and Col II compared to the Col-MA hydrogel-encapsulated NP-MTs (Fig. 6H–I), suggesting that the DNPM-MA hydrogel efficiently enhanced the deposition of NP matrix components.

### 3.5 The DNPM-MA hydrogel retarded the inflammaging of NP-MTs during tissue regeneration

**Fig. 7:**
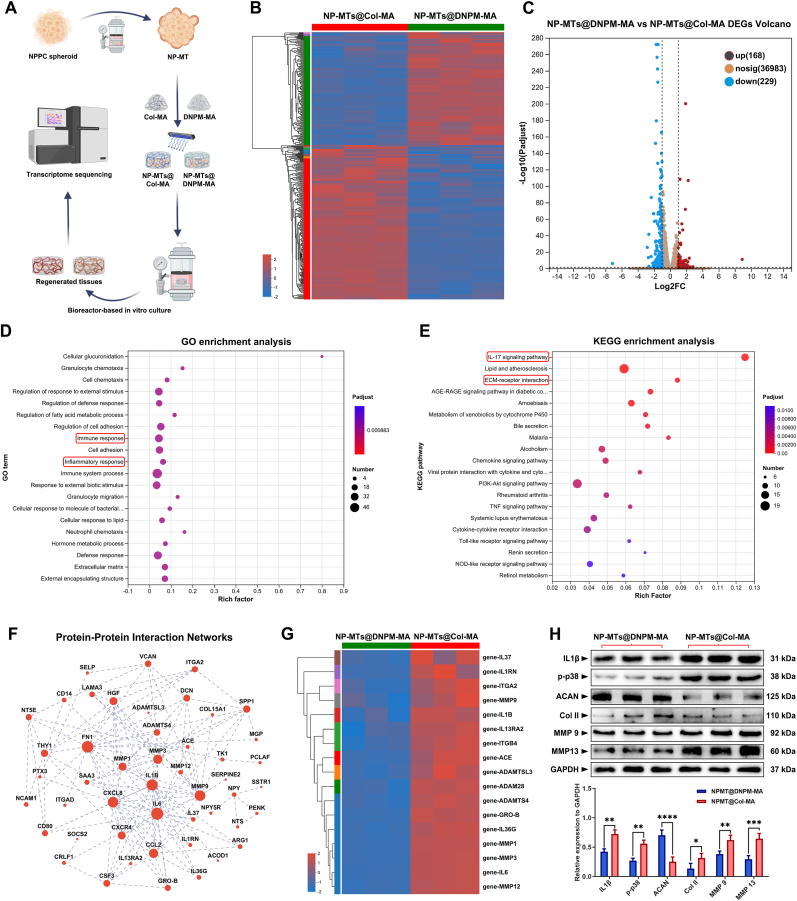
Molecular mechanisms underlying efficient NP regeneration induced by DNPM-MA-based TE-NP. (A) Schematic workflow of the RNA-seq procedure: Col-MA- and DNPM-MA-based TE-NPs were collected after 14 days of bioreactor culture for total RNA extraction, RNA-seq transcriptome library construction, DEG identification, and GO and KEGG enrichment analyses. (B–C) Heatmap (B) and volcano plot (C) showing the upregulated and downregulated DEGs in Col-MA-based TE-NP compared with DNPM-MA-based TE-NP. (D) GO enrichment analysis of the DEGs between Col-MA-based TE-NP and DNPM-MA-based TE-NP for total biological processes. (E) KEGG enrichment analysis of the DEGs between Col-MA-based TE-NP and DNPM-MA-based TE-NP for the corresponding pathways. (F) Protein-protein interaction networks of the inflammation and ECM metabolism related DEGs between Col-MA-based TE-NP and DNPM-MA-based TE-NP. (G) Heatmaps of biological processes involving inflammation and ECM metabolism from the corresponding GO and KEGG enrichment analyses. (H) Western blot analysis of the expression levels of inflammation and ECM metabolism related proteins in the Col-MA-based TE-NP and DNPM-MA-based TE-NP. ∗p < 0.05, ∗∗p < 0.01, ∗∗∗p < 0.001, and ∗∗∗∗p < 0.0001 were considered to indicate statistical significance.

To further explore the potential molecular mechanisms involved in the regeneration process induced by the DNPM-MA hydrogel, RNA sequencing analysis was performed to compare the differences in transcriptomes between the in vitro cultured NP-MTs@Col-MA and NP-MTs@DNPM-MA (Fig. 7A). The statistics of up- and downregulated differentially expressed genes (DEGs) in NP-MTs@DNPM-MA compared with NP-MTs@Col-MA are shown in the heatmap (Fig. 7B) and volcano plot (Fig. 7C). Significantly, as indicated by the GO analysis, the DEGs between the two types of TE-NPs were primarily involved in biological processes associated with regulating immune and inflammatory responses (Fig. 7D). Furthermore, Kyoto Encyclopedia of Genes and Genomes (KEGG) analysis revealed the enrichment of several pathways closely linked to inflammatory responses and ECM metabolism, including the IL-17 signaling pathway and ECM receptor interaction (Fig. 7E). As shown in Fig. 7F, the protein-protein interaction network highlighted that the DEGs with significant relevance were predominantly associated with inflammatory cytokines (e.g., IL1β, IL6, and IL37) and extracellular matrix catabolism-related factors (e.g., MMP3, MMP12, and MMP9). Moreover, a heatmap of the clustered inflammatory cytokines and ECM catabolism biomarkers revealed elevated expression in the NP-MTs@Col-MA group (Fig. 7G). Based on clues provided by the RNA sequencing analysis, a critical pathological alteration associated with inflammaging, an emerging concept of the cellular senescence-related inflammatory response [41], plays a key role in regulating the inflammatory response and ECM metabolism of DNPM-MA hydrogel-encapsulated NP-MTs. Consequently, inflammaging-related biomarkers were investigated by performing several biological experiments to further validate the molecular mechanical cues provided by the RNA-seq analysis.

**Fig. 8:**
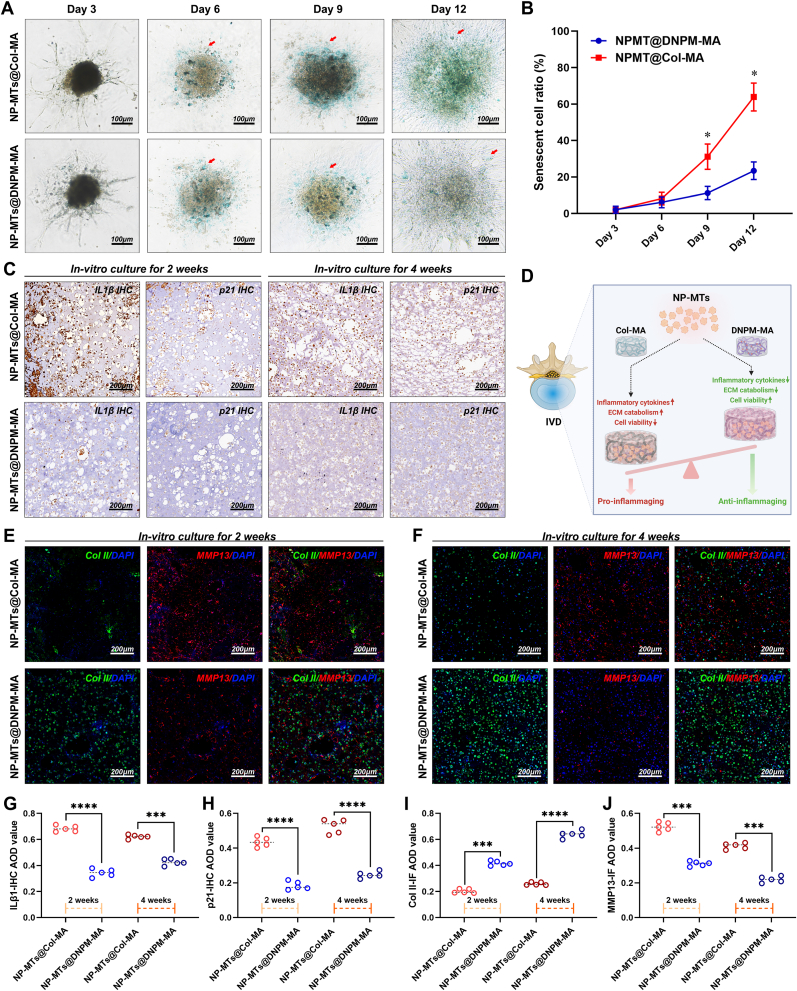
Assessment of inflammaging-related biomarkers in bioreactor-cultured TE-NPs. (A) Representative images of SA-β-gal staining of Col-MA and DNPM-MA hydrogel-encapsulated NP-MTs. (B) Statistical analysis of the SA-β-gal-positive cell ratio. (C) Representative images of IL1β and p21 IHC staining of Col-MA and DNPM-MA hydrogel-encapsulated NP-MTs cultured in a bioreactor for 2 and 4 weeks. (D) Schematic diagram of the underlying mechanism involved in DNPM-MA hydrogel-induced NP regeneration. (E) Representative images of Col II and MMP 13 IF staining of the Col-MA and DNPM-MA hydrogel-encapsulated NP-MTs cultured in a bioreactor for 2 weeks. (F) Representative images of Col II and MMP 13 IF staining of Col-MA and DNPM-MA hydrogel-encapsulated NP-MTs cultured in a bioreactor for 4 weeks. (G–H) Statistical analysis of the IL1β (G) and p21 (H) IHC AOD values of the Col-MA and DNPM-MA hydrogel-encapsulated NP-MTs cultured in a bioreactor for 2 and 4 weeks. (I–J) Statistical analysis of the Col II (I) and MMP13 (J) fluorescent AOD values of the Col-MA and DNPM-MA hydrogel-encapsulated NP-MTs cultured in a bioreactor for 2 and 4 weeks. ∗p < 0.05, ∗∗p < 0.01, ∗∗∗p < 0.001, and ∗∗∗∗p < 0.0001 were considered to indicate statistical significance.

As illustrated in Fig. 7H, the expression levels of IL1β, a hallmark inflammatory cytokine, and p-p38, a senescence-associated biomarker, were reduced in NP-MTs encapsulated in the DNPM-MA hydrogel after two weeks of in vitro culture. Conversely, the expression of the NP matrix components ACAN and Col II was markedly increased in NP-MTs encapsulated in the DNPM-MA hydrogel compared to those encapsulated in the Col-MA hydrogel. Additionally, the expression of the ECM catabolism biomarkers MMP9 and MMP13 was repressed in the regenerated tissues derived from NP-MTs@DNPM-MA compared with those derived from NP-MTs@Col-MA (Fig. 7H). SA-β-gal staining was subsequently performed to detect the degree of senescence of the NP-MTs loaded in the Col-MA and DNPM-MA hydrogels. Both the proportion of SA-β-gal-positive cells and the staining intensity were notably increased in NP-MTs encapsulated in Col-MA hydrogel compared to those seeded in the DNPM-MA hydrogel (Fig. 8A–B). Specifically, the slope of the culture duration-senescent cell ratio curve for the NP-MTs encapsulated in the Col-MA hydrogel was obviously steeper than that for the NP-MTs encapsulated in the DNPM-MA hydrogel, indicating that DNPM-MA could ameliorate the cellular senescence process of the NP-MTs (Fig. 8B). Furthermore, immunohistochemistry staining revealed that the expression of the inflammaging-associated biomarkers IL1β and p21 was markedly reduced in the regenerated tissues derived from NP-MTs@DNPM-MA compared to those derived from NP-MTs@Col-MA (Fig. 8C–G-H). Consistently, the immunofluorescence staining results also indicated that Col II expression was upregulated in the regenerated tissues derived from NP-MTs@DNPM-MA compared to those derived from NP-MTs@Col-MA (Fig. 8E–F, I), whereas MMP13 expression was significantly decreased in the regenerated tissues derived from NP-MTs@DNPM-MA compared to those derived from NP-MTs@Col-MA (Fig. 8E–F, J). In summary, the aforementioned findings from the evaluations at both the microtissue and engineered tissue levels collectively elucidated that the DNPM-MA hydrogels possessed biological effects on alleviating the inflammaging process and restoring the ECM metabolic homeostasis of the loaded NP-MTs, which helped foster a conducive intradiscal microenvironment for TE-NP transplantation-based tissue regeneration (Fig. 8D).

**Fig. 9:**
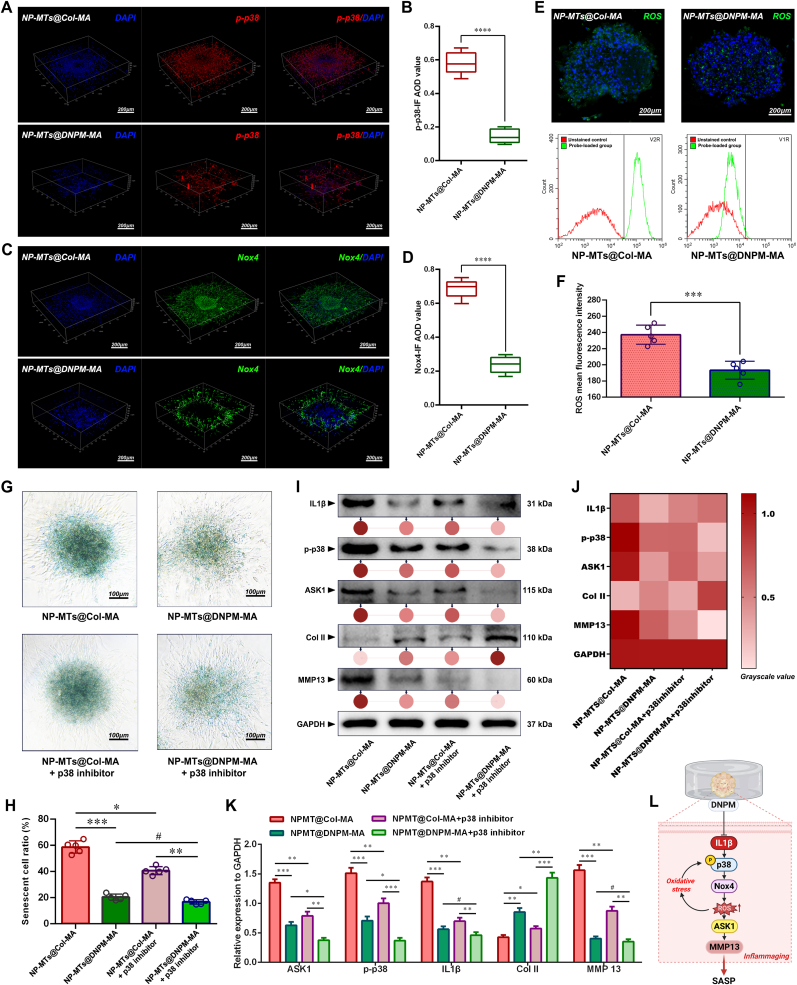
Assessment of p38/Nox4-driven SASP in bioreactor-cultured TE-NPs. (A) Representative p-p38 IF staining images of Col-MA and DNPM-MA hydrogel-encapsulated NP-MTs after 10 days of bioreactor culture. (B) Statistical analysis of the p-p38 fluorescent AOD values. (C) Representative Nox4 IF staining images of Col-MA and DNPM-MA hydrogel-encapsulated NP-MTs after 10 days of bioreactor culture. (D) Statistical analysis of the Nox4 fluorescent AOD values. (E) Observation and Flow cytometric quantification of intracellular ROS levels using DCFH-DA assay in Col-MA and DNPM-MA hydrogel-encapsulated NP-MTs after 10 days of bioreactor culture. (F) Statistical analysis of the ROS mean fluorescence intensity values. (G) Representative SA-β-gal staining images of NP-MTs with or without p38 inhibitor treatment after 10 days of bioreactor culture. (H) Statistical analysis of the SA-β-gal-positive cell ratio. (I) Western blot analysis of inflammaging- and ECM metabolism-related proteins in NP-MTs with or without p38 inhibitor treatment after 10 days of bioreactor culture. (J) Heatmap of the Western blot grayscale values. (K) Quantification of inflammaging- and ECM metabolism-related protein expression levels. (L) Schematic diagram showing the proposed mechanism by which DNPM downregulates the IL1β/p38/Nox4 mediated inflammaging axis, thereby reducing oxidative stress, SASP activation, and matrix catabolism of TE-NPs. **^#^**p > 0.05 was considered not statistically significant. ∗p < 0.05, ∗∗p < 0.01, ∗∗∗p < 0.001, and ∗∗∗∗p < 0.0001 were considered to indicate statistical significance.

To elucidate the underlying molecular mechanisms, RNA-seq analysis was conducted on NP-MTs encapsulated in DNPM-MA versus Col-MA hydrogels. Transcriptomic profiling revealed significant enrichment of the IL-17 signaling pathway and ECM-receptor interaction pathway in the DNPM-MA group (Fig. 7D–F), suggesting that the hydrogel microenvironment modulates inflammatory and matrix-associated gene expression programs. Among the key downstream effectors, p38 MAPK emerged as a central regulatory node, known to orchestrate IL1β and MMP expression, oxidative stress responses, and the senescence-associated secretory phenotype (SASP) in the context of intervertebral disc degeneration [42,43]. To validate this pathway, immunofluorescence staining demonstrated reduced expression of p-p38 (Fig. 9A–B) and NADPH oxidase 4 (Nox4), a major ROS producer upstream of p38 activation (Fig. 9C–D), in NP-MTs encapsulated in DNPM-MA compared to Col-MA. This suppression of oxidative stress was further confirmed by DCFH-DA staining, which revealed significantly lower ROS levels in DNPM-MA-encapsulated NP-MTs, while unstained controls were used to define the negative population and the same gating threshold was consistently applied across all samples (Fig. 9E–F). Given the pivotal role of p38 MAPK in inflammaging and matrix degradation, we employed SB203580, a selective p38 inhibitor, to determine whether DNPM-MA's protective effects are mediated via inhibition of this pathway. As shown in Fig. 9G–H, treatment of p38 inhibitor significantly alleviated cellular senescence of NP-MTs in Col-MA hydrogels, whereas its addition to DNPM-MA hydrogels conferred no further benefit, suggesting that p38 MAPK signaling is already suppressed in NP-MTs cultured in DNPM-MA (Fig. 9I–K). Consistently, western blot analysis revealed upregulated expression of p-p38, MMP13, and inflammaging-related proteins (IL1β and ASK1) in NP-MTs cultured in Col-MA, which were substantially suppressed upon p38 inhibition (Fig. 9I–K). In contrast, these markers were already downregulated in the DNPM-MA group, and p38 inhibition produced only marginal additional effects (Fig. 9I–K). Collectively, these results suggest that DNPM-MA attenuates NP-MT inflammaging and matrix catabolism, at least in part, via suppression of the p38 MAPK signaling cascade (Fig. 9L).

### 3.6 The DNPM-MA hydrogel promoted the regenerative efficiency of NP-MTs in vivo

**Fig. 10:**
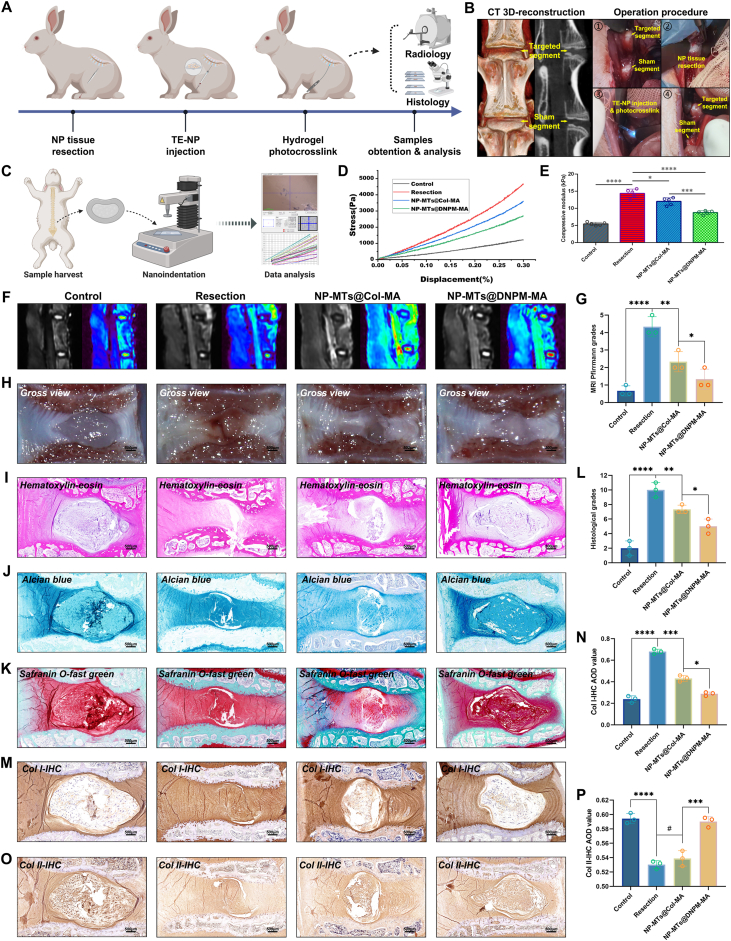
TE-NP implantation and evaluation of its tissue regeneration capacity in vivo. (A) Schematic illustration of rabbit lumbar intervertebral NP resection and TE-NP in situ implantation procedures. (B) The left photographs of CT 3D-reconstruction images revealed the sham and targeted intervertebral segments; and the right photographs revealed the NP resection and TE-NP implantation operation procedures. (C) Schematic illustration of rabbit lumbar intervertebral NP resection and nanoindentation-based compressive modulus testing procedures. (D–E) Nanoindentation-based stress-train (D) and compressive modulus analysis (E) of the rabbit lumbar intervertebral NP tissues after different surgical procedures. (F) Representative images of T2-weighted MRIs of the intervertebral segments and signal analysis. (G) Statistical analysis of changes in the Pfirrmann grade based on MRI of rabbit lumbar intervertebral NP tissues after different surgical procedures. (H–K) Representative images of the gross view (H) and histological assessments (including HE (I), Alcian blue (J), and Safranin O-fast green (K) staining of the intervertebral disc sections. (L) Statistical analysis of changes in the histological degenerative classification score of rabbit lumbar intervertebral NP tissues after different surgical procedures. (M − N) Col I IHC staining (M) and statistical analysis of the Col I-IHC AOD value (N) of rabbit intervertebral discs after different surgical procedures. (O–P) Col II IHC staining (O) and statistical analysis of the Col II-IHC AOD value (P) of rabbit intervertebral discs after different surgical procedures. **^#^**p > 0.05 was considered not statistically significant. ∗p < 0.05, ∗∗p < 0.01, ∗∗∗p < 0.001, and ∗∗∗∗p < 0.0001 were considered to indicate statistical significance. (For interpretation of the references to colour in this figure legend, the reader is referred to the Web version of this article.)

Ultimately, evaluating whether intradiscal transplantation of NP-MTs@DNPM-MA can effectively enhance the efficiency of NP regeneration in vivo is crucial to further elucidate the clinical and translational potential of this novel tissue engineering strategy. Consequently, a rabbit nucleotomy model was established to assess the in vivo regenerative efficacy of the two types of TE-NP. After performing the nucleotomy and TE-NP transplantation operations for 12 weeks, radiological and histological evaluations of the targeted IVDs were conducted (Fig. 10A). As illustrated in the left panels of Fig. 10B, in the CT 3D-reconstruction image, the IVD between the third and fourth lumbar vertebrae was selected as the targeted segment for the operation, while the IVD between the fourth and fifth lumbar vertebrae was designated the sham segment for the control. The right panels in Fig. 10B show the operating procedure, which included: a. exposure of the IVDs through an anterior approach; b. excision of the NP tissues within the target segment; c. Implantation of the NP-MT-encapsulated hydrogel precursor; d. crosslinking of the hydrogels under ultraviolet fiber optic irradiation; and e. subsequent closure of the surgical incision. Moreover, in vivo experimental (L3/L4 segment) and sham (L4/L5 segment) comparisons were performed within the same animal to minimize inter-individual variability. Full-field strain analysis and regional stress–strain curves under mechanical loading, obtained by digital image correlation (DIC), further confirmed that adjacent IVD segments exhibited no significant biomechanical differences (Fig. S7).

Additionally, a depiction of the operating procedure used for coronal sectioning is provided in Fig. S8 of the Supplementary Material and Video, indicating that the injectable TE-NP, composed of photocrosslinkable DNPM-MA hydrogel-encapsulated NP-MTs, could effectively fill the NP cavity and crosslink to form a gel-like structure analogous to that of natural NP tissue. To assess the mechanical properties of the regenerated NP tissue in vivo, compressive modulus testing was performed using a nanoindentation-based stress–strain analysis (Fig. 10C–D). As shown in Fig. 10E, the resection group exhibited the highest compressive modulus (14.83 ± 1.67 kPa), followed by the NP-MTs@Col-MA group (12.06 ± 0.96 kPa). In contrast, the NP-MTs@DNPM-MA group displayed a significantly lower modulus (8.65 ± 1.13 kPa), approaching that of the control group (5.32 ± 0.81 kPa). These findings suggested that while DNPM-MA hydrogel facilitated NP tissue regeneration, the mechanical property of the regenerated tissue was closer to that of the native NP tissues in control group than to that achieved with Col-MA or post-resection healing.

A radiological evaluation using magnetic resonance imaging (MRI) showed that the hydration status of NP tissue was correlated with the severity of disc degeneration. An analysis of the MRI signals revealed that the implantation of NP-MTs@Col-MA and NP-MTs@DNPM-MA alleviated the progression of NP degeneration to a certain extent, but the tissue rehydration effect induced by the transplantation of NP-MTs@DNPM-MA markedly exceeded that induced by the transportation of NP-MTs@Col-MA (Fig. 10F–G). The sagittal gross view of the harvested IVDs further indicated that the regenerated tissues derived from NP-MTs@DNPM-MA were most similar in range and morphology to the healthy NP tissues in the control group, which presented as a pale white gel-like substance (Fig. 10H). Additionally, histological assessments including HE (Fig. 10I), Alcian blue (Fig. 10J), and Safranin O-fast green (Fig. 10K) staining, also suggested that implantation of the TE-NP constructed of NP-MTs@DNPM-MA exhibited superior NP regeneration effects compared to those of NP-MTs@Col-MA (Fig. 10L), according to the histopathology scoring system for disc degeneration in animal models [44,45]. As the primary ECM structural component of NP tissue, alternations in the collagen composition within the NP tissues were analyzed using IHC staining, which collectively confirmed that the implantation of NP-MTs@DNPM-MA was advantageous for mitigating tissue fibrosis by inhibiting Col I synthesis (Fig. 10M,N) and fostering Col II deposition (Fig. 10O–P).

## 4 Discussion

The core gel-like NP, which is enveloped by the upper and lower cartilage endplates and surrounded by the annulus fibrosus, acts as the primary load-bearing and cushioning component of IVDs [2,4,38]. The accumulation of senescent cells and deterioration of the ECM in the NP are the primary pathological features of the progression of IVD degeneration [17,46]. Although biological therapies, including cell transplantation, growth factor treatment, or gene therapy, have showed efficacy in addressing DDD, the pathogenic conditions within the IVD, such as abnormal inflammatory, mechanical, and oxidative stresses, still lead to a high rate of cellular senescence and mortality [30,47]. Moreover, an imbalance in functional ECM metabolic homeostasis can further impact the normal biological behavior of cells and impair the biomechanical function of NP tissue, ultimately inducing and aggravating the vicious cycle of disc degeneration [17,48,49]. Tissue engineering and regenerative medicine approaches have long been considered promising medical interventions due to their capacity to repair, enhance, or replace tissues or organs that exhibit dysfunctional attributes stemming from trauma, stress injury, or aging [5,7,50]. Hence, inhibiting cell senescence and restoring functional ECM metabolic homeostasis are not only the objectives of NP repair or regeneration, but also the fundamental prerequisites for developing TE-NP.

In traditional tissue engineering, the "top-down' approach involves seeding cells onto a prefabricated 3D biodegradable scaffold with the expectation that they will attach, proliferate, and eventually fully populate the scaffold while simultaneously depositing newly formed ECM over time [51,52]. However, immobilizing cells within preformed ECM-mimetic biomaterials is highly challenging, often leading to low cell seeding density and uneven spatial distribution [53,54]. Additionally, the lack of a natural ECM environment significantly limits the bioactivity and adaptability of isolated seeded cells in a biomaterial-based microenvironment. These limitations, coupled with an increasing understanding of human developmental biology, have supported the bioinspired "bottom-up' creation of 3D seed units (e.g. cell aggregates/spheroids, microtissues, or organoids) [55,56]. Notably, self-assembled 3D cellular spheroids have rapidly emerged as appealing cell-rich unitary building blocks for replicating in vivo organ functional units since 3D multicellular aggregates with spherical shapes are also observed during tissue morphogenesis [36,53]. Previous studies have shown that spheroidal cell niches are present in NP tissues, and these spheroidal cell clusters exhibit a greater capacity for self-renewal and ECM synthesis than adhesive cell clusters [46,57]. Guided by "bottom-up' tissue engineering principles and the histological characteristics of human and animal NP tissues, we previously developed an injectable TE-NP composed of hydrogel-encapsulated cell spheroids for intradiscal implantation [9]. Although the transplantation of this TE-NP enhances the regeneration of NP to a certain extent, some shortcomings have also been observed [9]. First, in this TE-NP, the cell aggregates have not yet formed an abundant pericellular matrix. After being encapsulated by hydrogels, the cells within aggregates still need to adapt to the supporting material, which in turn affects their normal phenotype and bioactivity. Second, the bioscaffold used for TE-NP construction was a gelatin-based photocrosslinkable hydrogel. While this hydrogel exhibits favorable structural and mechanical biomimetic properties, it still displays considerable disparities in functional components compared to the natural NP matrix.

In light of the previously mentioned limitations, the present research employed an in vitro biomimetic cultivation approach to generate NP-MTs with nascent ECM surroundings, which could serve as seed and regenerative units in place of cell aggregates. Concurrently, a novel photocrosslinkable hydrogel fabricated based on chemical modification of DNPM was utilized as the supporting biomaterial for TE-NP construction. Through a dual biomimetic design that considered both functional components and mechanical characteristics, the newly developed TE-NP further augmented the effects of the functional ECM on sustaining the biological activities of the seed cells, thereby improving the efficacy of the TE-NP in facilitating NP regeneration. NPPCs have been extensively applied in TE-NP construction and IVD regeneration research, owing to their intrinsic phenotypic stability, ability to secrete NP-specific ECM components such as ACAN and Col II, and responsiveness to mechanical and biochemical cues that replicate the native disc environment [25,58]. Specifically, NP-MTs in this study were cultivated from NPPCs due to their well-documented capacity to adapt to the unique microenvironment within NP tissue and their multifaceted potential in promoting tissue repair and regeneration [23,59]. Recent high-impact studies, including single-cell transcriptomic analyses and spatiotemporal characterizations, have confirmed the presence of NPPCs with stem/progenitor-like properties within IVD cell niches [13,37]. These cells demonstrate the ability to undergo multilineage differentiation, including osteogenic, chondrogenic, and adipogenic lineages, and are characterized by the positive expression of stem/progenitor cell markers such as CD73 and CD105, alongside the negative expression of hematopoietic and immune-related markers such as CD34 and HLA-DR [25,37]. Given their strong regenerative capacity, phenotypic stability, and adaptability to the harsh NP microenvironment, NPPCs were chosen as the seed cells for NP-MT construction, providing a highly promising therapeutic strategy for the treatment of DDD.

Moreover, as a primary compression-buffering structure of IVD, the NP is highly hydrated and gelatinous, and its functional ECM components are mainly composed of hydrophilic proteoglycans (mostly ACAN, constituting approximately 50 % of the dry weight), and Col II (constituting approximately 20 % of the dry weight) [60]. Hence, the cells within NP tissue reside in a microenvironment with continuous hydrostatic pressure, which is mediated by a composition of approximately 80–90 % water in the matrix under axial compression [[60], [61], [62]]. The hydrostatic pressure within the healthy NP cavity of adults remains at a low magnitude between 0.12 and 0.45 MPa when in a supine or sitting position [63]. Our previous research also revealed that low magnitude hydrostatic pressure (less than or equal to 0.5 MPa) was conducive to sustain cellular viability and the metabolic homeostasis of functional ECM components in NP tissues [12]. Therefore, in the present study, low-magnitude hydrostatic pressure loading was applied during the cultivation of NP-MTs using our custom-designed hydrostatic bioreactor. The results showed that a hydrostatic pressure of 0.2 MPa was the optimal parameter for maintaining cell activity and functional ECM deposition within the microtissues. Although the critical molecular mechanisms involved in the process of NP-MT cultivation require further investigation, this novel bioreactor-based scheme yielded microscale seed units for constructing TE-NPs with high cellular activity and abundant functional matrix encapsulation. It also provides a promising choice of microscale regenerative units to generate engineered NP analogs in a “bottom-up” manner.

Hydrogel is a 3D network structure composed of crosslinkable hydrophilic polymers [64]. A hydrogel can absorb up to thousands of times its weight in water and can be fabricated into injectable forms, closely mimicking the natural properties of the NP matrix [65,66]. In addition to good biocompatibility and mechanical properties, the ideal design of a hydrogel for constructing TE-NP should have the potential to sustain cellular activity and functional ECM deposition [67,68]. However, current natural or synthetic hydrogels still have various limitations in replicating both the structural characteristics and componential properties of native NP tissues, which are unfavorable for NP repair and regeneration [30,66,69]. Hence, we speculate that modifying natural DNPM into a photocrosslinkable hydrogel would be more suitable for the construction of TE-NP, due to its dual bionic properties in terms of physical and biological characteristics [[28], [29], [30]]. Our findings demonstrated that DNPM-MA possessed a tunable elastic modulus, making it well-suited for constructing TE-NP with mechanical properties closely resembling those of native NP tissue. Its degradation rate was well-matched to the pace of tissue regeneration, and it exhibited superior mechanical performance in regenerated tissues both in vitro and in vivo. Furthermore, the excellent injectability of DNPM-MA greatly broadened its potential clinical applications, aligning seamlessly with current minimally invasive treatment paradigms for DDD. The clinical application of xenogeneic decellularized matrix materials has become increasingly widespread [70,71]. However, potential risks persist, including residual immunogenicity and the theoretical possibility of disease transmission [71]. To mitigate these risks, rigorous decellularization and sterilization procedures are routinely employed. Reports of immunogenicity are largely attributed to residual genetic material from incomplete decellularization [71,72]. Notably, recent advances in comprehensive material modification and processing—such as chemical grafting, electrospinning, and 3D printing—have further reduced immune responses and the risk of graft rejection [70,73]. In the present study, we adopted a combined enzymatic and chemical decellularization approach, followed by MA modification, which markedly decreased the risk of residual immunogenicity. Nevertheless, future studies incorporating more rigorous immunological assessments will be exerted to fully validate the biosafety of DNPM-MA and support its clinical translation.

The outcomes of the current in vitro and in vivo assessments of the engineered NP's regenerative process indicated that TE-NP constructed from NP-MTs@DNPM-MA exhibited superior tissue regeneration effects compared to TE-NP constructed from NP-MTs@Col-MA. Specifically, histological assessments revealed a more uniform distribution of cells and matrix within the NP-MTs@DNPM-MA-derived regenerated tissues. Moreover, increased synthesis of functional ECM components and decreased expression of catabolic metabolism-associated biomarkers were observed in NP-MTs@DNPM-MA-derived regenerated tissues. MRI analysis of the IVDs also showed that the targeted NP segment subjected to intradiscal transplantation of NP-MTs@DNPM-MA had a higher water content than that subjected to intradiscal transplantation of NP-MTs@Col-MA. Taken together, these findings elucidated that the TE-NP constructed from NP-MTs@DNPM-MA could serve as a promising graft to promote NP regeneration both in vivo and in vitro. Importantly, in our study, sham and experimental groups were assigned to adjacent discs within the same rabbit, thereby minimizing inter-individual variability. Given the reported inherent differences in hydrophilic constituent content among rabbit NP tissues [74], the use of self-adjacent discs provided a more accurate internal control. Specifically, the L3/L4 segment served as the treatment group and the L4/L5 segment as the sham group. In non-bipedal animals such as rabbits, low spinal axial loading and strong facet joint stabilization reduce stress disparities between neighboring segments, and prior studies have demonstrated no significant differences in the mechanical properties of L3/L4 and L4/L5 discs [75,76]. Consistently, our DIC-based full-field strain analysis and regional stress–strain curves further confirmed comparable biomechanical behavior, supporting the conclusion that adjacent segments share similar mechanical environments and are unlikely to confound experimental outcomes.

Furthermore, an RNA sequencing-based transcriptome analysis was subsequently performed to elucidate potential regulatory pathways that account for the distinct biological behaviors observed between the DNPM-MA hydrogel-encapsulated NP-MTs and Col-MA hydrogel-encapsulated NP-MTs. This approach identified several enriched pathways related to inflammatory responses and ECM catabolism, which suggested a significant correlation between cellular senescence of the NP-MTs and the bioactive properties of the DNPM-MA hydrogel. Inflammation serves as a dual-faceted entity in the biological realm: it activates the body's defensive mechanisms to counteract invading pathogens and plays a pivotal role in the natural healing process following injury, while it also possesses the propensity to elicit undesirable or unrestrained reactions [77]. These latter responses can culminate in tissue damage and functional anomalies, underscoring its paradoxical nature [77,78]. Aging, which is typified by a gradual deterioration of interconnected physiological systems and metabolic pathways, can further undermine the body's immune system, rendering it more susceptible to deleterious inflammatory reactions [77,78]. One critical hallmark of aging is cellular senescence, which has been extensively examined as a pathological indicator of DDD [79]. Independent of the nature of the senescence trigger, senescent cells manifest a "senescence-associated secretory phenotype' (SASP) characterized by the secretion of proinflammatory cytokines, chemokines, growth factors, and matrix metalloproteases (MMPs) [80,81]. Numerous studies have established a correlation between the cellular SASP and the progression of disc degeneration [79,80]. Additionally, pathological factors within the microenvironment of degenerative IVDs, such as mechanical, oxidative, and inflammatory stresses, stimulate the production, accumulation, and excessive expression of SASP components [[82], [83], [84]]. Inflammaging, an emergent concept that emphasizes a senescence-linked chronic inflammatory response, has become an emerging research hotspot due to its implications for numerous degenerative diseases [77,78]. A recent study elucidated that eliminating proinflammaging stimuli within the intradiscal microenvironment achieved effective IVD regeneration outcomes [85]. The interplay between ECM metabolism and cellular senescence forms a reciprocal loop, where the ECM can signal and regulate the behaviors of senescent cells, while senescent cells, in turn, influence the composition and structure of the ECM via their secretome [41,81,84]. This secretome encompasses various MMPs, which may lead to alternative ECM properties and subsequently aggravate disc degeneration [84].

Among the signaling pathways involved, p38 MAPK serves as a critical regulator of proinflammatory cytokine expression, matrix degradation, and senescence. In DDD models, p38 MAPK activation induces IL-1β and MMP expression, reinforcing the SASP phenotype. To investigate whether DNPM-MA hydrogel exerts effects via this pathway, we applied a selective p38 MAPK inhibitor. Inhibition of p38 significantly decreased p-p38, IL-1β, and MMP13 levels, while increasing ACAN and Col II expression. Notably, DNPM-MA treatment alone produced comparable effects to p38 inhibition, and combined treatment did not yield additive benefits, indicating DNPM-MA's anti-inflammatory and regenerative actions are mediated, at least partly, through suppression of p38 MAPK signaling [27,42]. Supporting this, DNPM-MA reduced SA β-gal activity, proinflammatory cytokine expression, and matrix catabolism, collectively indicating an attenuation of inflammaging in our study. Our findings further suggested that DNPM-MA hydrogels reconstructed the NP-MT microenvironment, protecting cells from inflammaging-associated chemokines and MMPs, thereby mitigating SASP progression and enhancing functional ECM secretion. Mechanistically, IL1β acts upstream by activating p38 MAPK, which upregulates Nox4, a key ROS source in NPPCs [86]. Elevated ROS induces oxidative stress, activates ASK1, and further promotes MMP13 expression, establishing a positive feedback loop sustaining SASP. DNPM appears to suppress IL1β at this upstream level, disrupting the inflammatory-oxidative cycle, reducing catabolic enzyme production, and ultimately mitigating disc inflammaging. This mechanism underscores DNPM-MA's potential as a therapeutic platform for delaying or reversing degenerative changes in DDD.

Although the current work provides innovative and significant findings that advance "bottom-up' tissue engineering-based therapies for treating DDD, some limitations also exist. Our research, for the first time, presented an NP-MT cultivation strategy using a novel self-developed hydrostatic bioreactor and revealed that low-magnitude hydrostatic pressure conditions were beneficial for the maintenance of cell viability and the accumulation of functional ECM components. Hence, we provided an optimized cultivation platform and methodology to generate bioactive seeding and regenerative units for the construction of TE-NP. However, the potential regulatory mechanisms involved in the cultivation of microtissues are quite complex and are related to both cell-to-cell and cell-to-ECM interactions, intracellular signaling, cytoskeletal reorganization, and ECM metabolism pathways. Further in-depth and comprehensive studies are still required regarding the construction methods, parameters, and regulatory mechanisms of long-term NP-MT cultivation processes, as well as the feasibility of achieving an organoid-like regenerative unit. Additionally, as a natural material derived from animal tissues, heterologous DNPM may carry the risk of viral infection. The composition, mechanical properties, and degradability of heterologous DNPM may also differ from those of human NP tissues. Therefore, testing the immunogenicity and biosafety of DNPM-MA hydrogels in more species and developing more appropriate decellularization methods for clinical application are important. In fact, we have initiated studies to explore various complementary decellularization techniques, such as the integration of physical and chemical methods using a bioreactor [20], aiming to optimally preserve the ECM structure and biological activity of these materials. Moreover, according to the transcriptomic and proteomic sequencing, along with subsequent biological experiments, we have preliminarily declared that the mechanism of DNPM-MA hydrogel-mediated NP regeneration is associated with mitigating p38 MAPK-associated inflammaging of the seed cells in NP-MTs. Nevertheless, the key components and regulatory pathways through which the DNPM-MA hydrogel exerts this biological effect warrant further elucidation in future studies.

## 5 Conclusion

To conclude, the present study demonstrated that the construction of TE-NP via a "bottom-up' approach using hydrostatic bioreactor-cultivated NP-MTs as seed units and a photocrosslinkable DNPM-MA hydrogel as a supporting scaffold yielded promising tissue regeneration outcomes both in vitro and in vivo. The regulatory mechanism involved in this regeneration process is intimately linked to the attenuation of the inflammaging intensity. On the one hand, based on the mechanobiological behaviors of the cells in NP-MTs, we introduced an innovative bioreactor-based cultivation procedure for producing microscaled seed units, which were initially encapsulated within the nascent matrix and subsequently exhibited good adaptability to the intradiscal microenvironment. Additionally, the DNPM-MA hydrogel contained natural ECM components such as Col II, GAGs, and trace growth factors, and was endowed with favorable mechanical properties due to the modification of photocrosslinkable methacrylate groups. The dual bionic design of both compositional and mechanical attributes of the hydrogel was beneficial for the growth, migration, proliferation, and ECM synthesis of the host seed units, positioning it an ideal biomaterial for supporting the long-term cultivation of NP-MTs. Taken together, this study proposes a pioneering seed unit and provides an ideal biomimetic hydrogel for TE-NP construction, providing insights into "bottom-up' tissue engineering-based therapies for NP regeneration.

## CRediT authorship contribution statement

**Xiaoxiao Li:** Writing – original draft, Methodology, Conceptualization. **Xiangwei Li:** Writing – original draft, Methodology. **Dandan Zhou:** Writing – original draft, Methodology, Data curation. **Yanqin Xu:** Methodology. **Biemin Sun:** Data curation. **Yanzhu Hu:** Software. **Yibo Zhu:** Methodology. **Junxian Hu:** Data curation. **Zeyu Pang:** Investigation. **Chen Zhao:** Investigation. **Yongjian Gao:** Investigation. **You Long:** Investigation. **Pei Li:** Project administration, Funding acquisition. **Qiang Zhou:** Supervision, Project administration, Funding acquisition. **Yiyang Wang:** Writing – review & editing, Project administration, Conceptualization.

## Generative AI statement

The authors declare that no Generative AI was used in the creation of this manuscript.

## Declaration of competing interest

The authors declare the following financial interests/personal relationships which may be considered as potential competing interests.

## Data Availability

Data will be made available on request.
