# Supplementary material for: Bottom-up engineering of the nucleus pulposus using a photocrosslinkable decellularized matrix hydrogel attenuates inflammaging and enhances microtissue-mediated regeneration
Source: Mater Today Bio. 2025 Sep 24;35:102347. doi: 10.1016/j.mtbio.2025.102347 (PMC12524782; doi:10.1016/j.mtbio.2025.102347)
Supplement: Multimedia component 1 [file mmc1.pdf]

Supplementary Material for

Bottom-Up Engineering of the Nucleus Pulposus Using a Photocrosslinkable Decellularized Matrix Hydrogel Attenuates Inflammaging and Enhances Microtissue-Mediated Regeneration

Xiaoxiao Li[1, 2,*], Xiangwei Li[1, 2,*], Dandan Zhou[3,*], Yanqin Xu[4], Biemin Sun[4], Yanzhu Hu[5], Yibo Zhu[1, 2], Junxian Hu[1, 2], Zeyu Pang[1, 2], Chen Zhao[1], Yongjian Gao[1], You Long[1], Pei Li[1, #], Qiang Zhou[1, 2, #], Yiyang Wang[1, 2, #]

[1] Department of Orthopedics, The Third Affiliated Hospital of Chongqing Medical University, Chongqing 401120, China

[2] Tissue Repairing and Biotechnology Research Center, The Third Affiliated Hospital of Chongqing Medical University, Chongqing 401120, China

[3] Department of Geriatric Medicine, Jiulongpo People's Hospital of Chongqing, Chongqing 400050, China

[4] College of Chemistry and Chemical Engineering, Chongqing University, Chongqing 400044, China

[5] Department of Surgery, TUM School of Medicine and Health, Klinikum rechts der Isar, Technical University of Munich, Munich 81675, Germany

[*] These authors contributed to the work equally and should be regarded as co-first authors

[#] Correspondence to: Yiyang Wang (Yee-spine@hospital.cqmu.edu.cn); Qiang Zhou (E-mail: zhouqiang@hospital.cqmu.edu.cn); Pei Li (lipei@hospital.cqmu.edu.cn)

Keywords: Nucleus pulposus progenitor cells (NPPCs), Photocrosslinkable hydrogel, Microtissue, Tissue engineered nucleus pulposus (TE-NP), Inflammaging

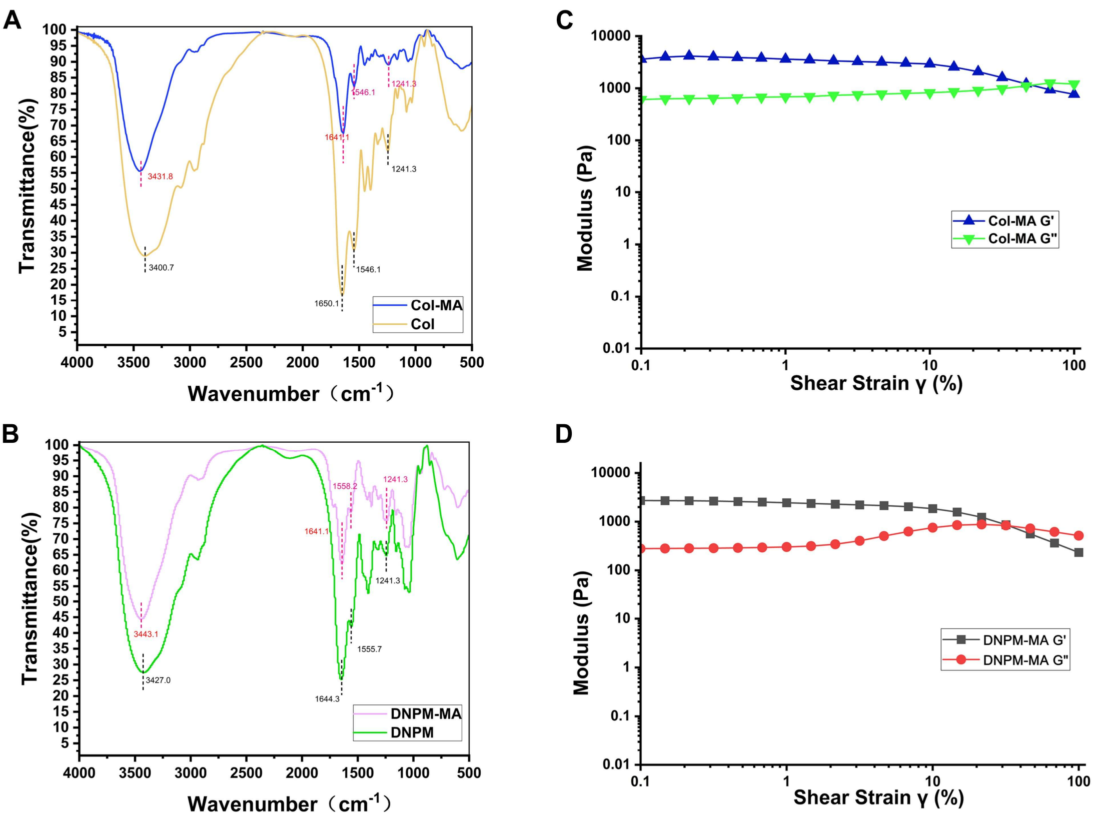

Fig. S1. Fourier Transform Infrared (FTIR) and rheological characterization of Col-MA and DNPM-MA hydrogels. A) FTIR spectrum of Col-MA hydrogel. B) FTIR spectrum of DNPM-MA hydrogel. C) Storage modulus (G′) and loss modulus (G″) of Col-MA hydrogel. D) The G′ and G″ of DNPM-MA hydrogel.

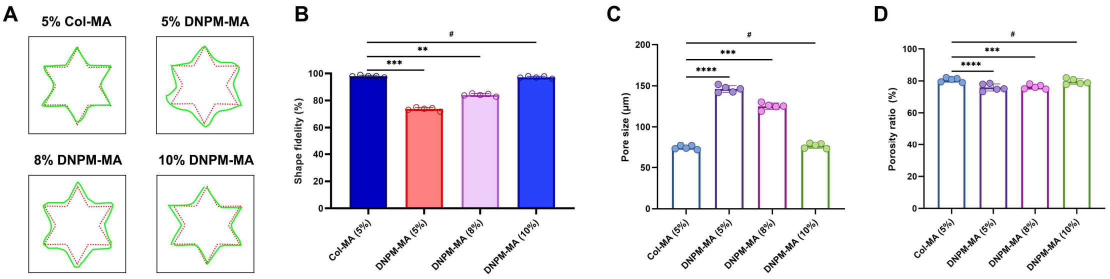

Fig. S2. Assessments of shape fidelity and porosity of Col-MA and DNPM-MA hydrogels. A) Comparison of outlines of crosslinked 5% Col-MA and 5%, 8%, and 10% DNPM-MA hydrogels demolded from a hexagonal star-shaped mold. B) Statistical analysis of the shape fidelity ratio of crosslinked 5% Col-MA and 5%, 8%, and 10% DNPM-MA hydrogels. C) Statistical analysis of the pore dimensions and D) porosity ratio of crosslinked 5% Col-MA and 5%, 8%, and 10% DNPM-MA hydrogels. $^{\#}p > 0.05$ was considered not statistically significant. $^{*}p < 0.05$, $^{**}p < 0.01$, $^{***}p < 0.001$, and $^{****}p < 0.0001$ were considered to indicate statistical significance.

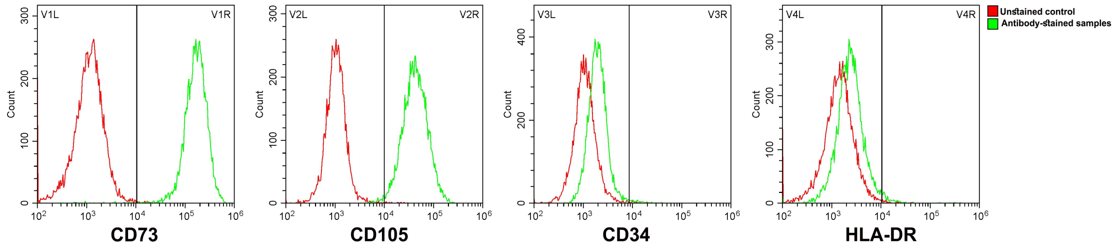

Fig. S3. Characterization of NPPC phenotype. Specific cell surface positive and negative markers of the NPPCs (CD73, CD105, CD34 and HLA-DR) were identified by the fluorescence activated cell sorter (FACS) using flow cytometry. Representative histograms illustrate marker expression. Unstained controls were included in each batch of analysis and used to define the boundaries between positive and negative cell populations, and the same threshold was consistently applied to all stained samples.

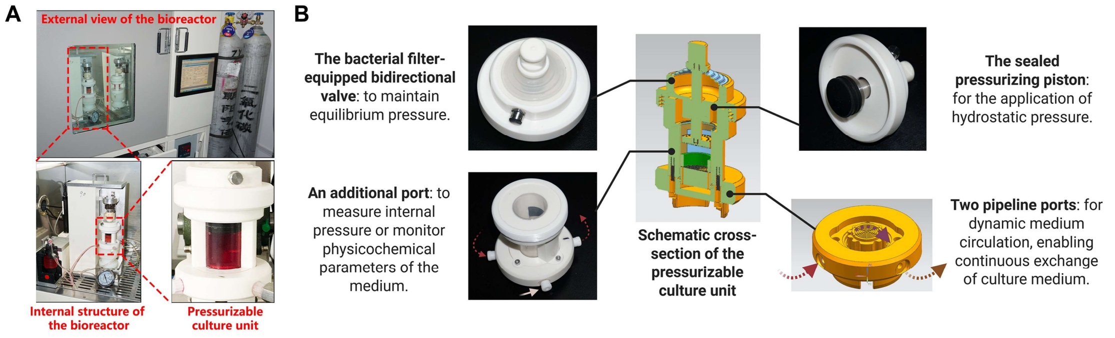

Fig. S4. The appearance, components, and internal structure of our self-developed hydrostatic bioreactor: a pictorial introduction. A) Appearance and key component of our self-developed hydrostatic bioreactor for cultivation of the NP-MTs and maturation of the in vitro cultured TE-NPs: Appearance of the tissue culture conditions operating and gas exchange operating system, Details of the hydrostatic pressure exerting and medium perfusing devices, and Appearance of the hydrostatic culture incubator, B) Descriptive illustration and operational mechanism of the internal structures of the hydrostatic culture incubator.

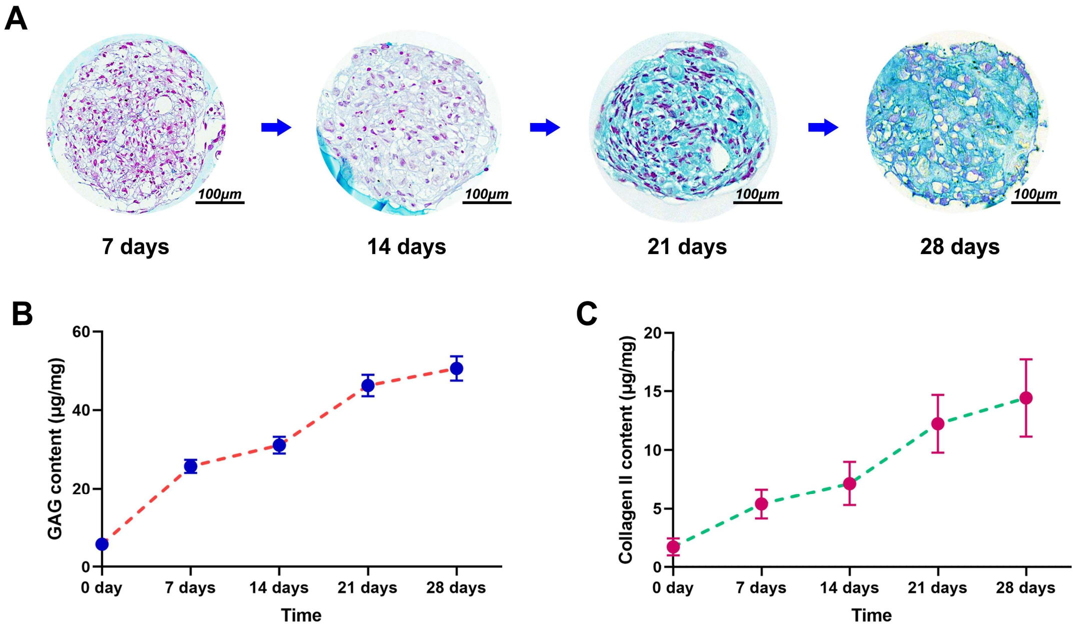

Fig. S5. Cultivation procedure and matrix component analysis of NP-MTs. A) Alcian blue staining of the in vitro cultured NP-MTs at defined time points. B) Quantification of GAG content of the in vitro cultured NP-MTs at define time points. C) Quantification of collagen II content of the in vitro cultured NP-MTs at define time points.

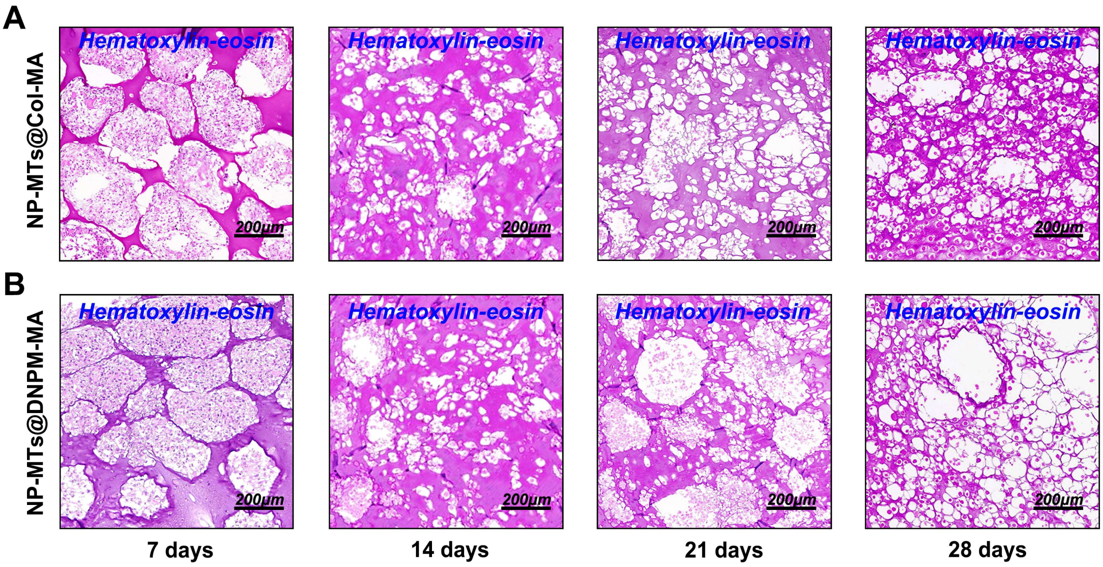

Fig. S6. Hydrostatic bioreactor-based TE-NP maturation. A) HE staining of in vitro cultured TE-NPs constructed using NP-MTs@Col-MA at defined time points. B) HE staining of in vitro cultured TE-NPs constructed using NP-MTs@DNPM-MA.

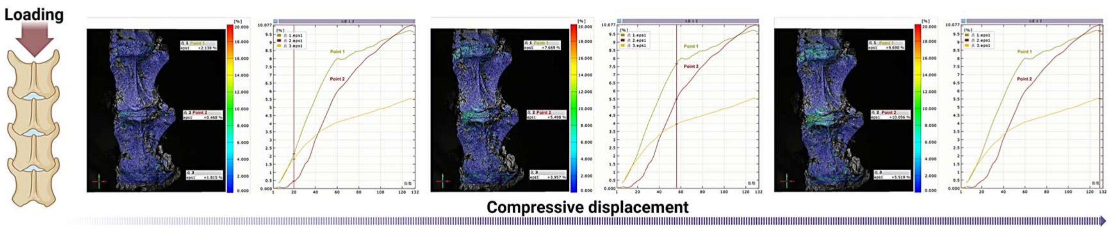

Fig. S7. Full-field strain analysis and regional stress–strain curves of adjacent IVDs under mechanical loading. Representative full-field strain maps obtained by digital image correlation (DIC) are shown at different time points during loading. Strain values were extracted from predefined anatomical points (point 1 and point 2), representing adjacent segments used as the sham and resection groups for further treatments. The resulting stress-strain curves indicate heterogeneous mechanical responses across regions; however, no significant biomechanical differences were observed between the adjacent segments under loading. These findings suggest that the mechanical environments of adjacent segments are comparable and unlikely to confound the experimental outcomes.

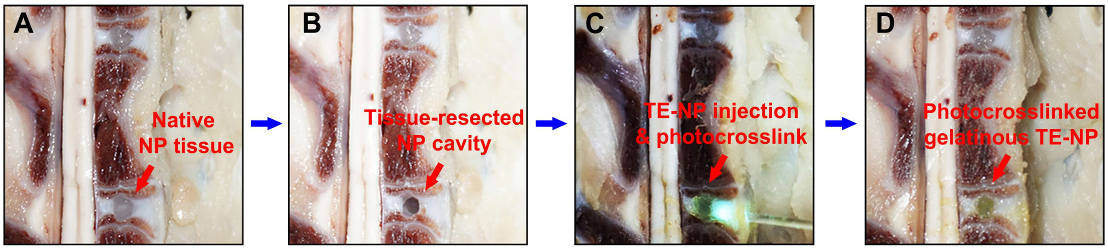

Fig. S8. Depiction of the TE-NP implantation procedure in a coronal view.
